# Divergence of Gene Body DNA Methylation and Evolution of Plant Duplicate Genes

**DOI:** 10.1371/journal.pone.0110357

**Published:** 2014-10-13

**Authors:** Jun Wang, Nicholas C. Marowsky, Chuanzhu Fan

**Affiliations:** Department of Biological Sciences, Wayne State University, Detroit, Michigan, United States of America; Michigan State University, United States of America

## Abstract

It has been shown that gene body DNA methylation is associated with gene expression. However, whether and how deviation of gene body DNA methylation between duplicate genes can influence their divergence remains largely unexplored. Here, we aim to elucidate the potential role of gene body DNA methylation in the fate of duplicate genes. We identified paralogous gene pairs from *Arabidopsis* and rice (*Oryza sativa* ssp. *japonica*) genomes and reprocessed their single-base resolution methylome data. We show that methylation in paralogous genes nonlinearly correlates with several gene properties including exon number/gene length, expression level and mutation rate. Further, we demonstrated that divergence of methylation level and pattern in paralogs indeed positively correlate with their sequence and expression divergences. This result held even after controlling for other confounding factors known to influence the divergence of paralogs. We observed that methylation level divergence might be more relevant to the expression divergence of paralogs than methylation pattern divergence. Finally, we explored the mechanisms that might give rise to the divergence of gene body methylation in paralogs. We found that exonic methylation divergence more closely correlates with expression divergence than intronic methylation divergence. We show that genomic environments (e.g., flanked by transposable elements and repetitive sequences) of paralogs generated by various duplication mechanisms are associated with the methylation divergence of paralogs. Overall, our results suggest that the changes in gene body DNA methylation could provide another avenue for duplicate genes to develop differential expression patterns and undergo different evolutionary fates in plant genomes.

## Introduction

Epigenetic modifications of DNA and histones can inheritably regulate the access to the genetic information encoded by DNA [1]–[4]. DNA methylation, defined as adding a methyl group to the cytosine base of DNA to form 5-methylcytosine, is one of such epigenetic modifications [5]. In plants, cytosine can be methylated in three sequence contexts, CG, CHG and CHH (H = A, C, or T) [6]. DNA methylation can be established *de novo* and maintained by various methyltransferases, e.g. DRM2, MET1, and CMT3 in plants [7]–[11]. It can also be removed either passively by malfunction of methylation maintenance pathway [12], or actively by demethylation enzymes, e.g. DME and ROS1 in plants [13], [14]. DNA methylation is involved in various important biological processes such as repressing the expression of TEs and repetitive elements, and participating in early embryogenesis, stem cell differentiation, X chromosome inactivation, genomic imprinting, neuronal and cancer development [15]–[22].

DNA methylation can occur in the promoter and gene body regions of genes, which influence gene expression differently. DNA methylation in promoter regions is usually negatively associated with gene expression [23], [24]. Whereas, in gene body extreme low or high DNA methylation level is associated with lower gene expression, while modest DNA methylation levels is related to higher expression [25]–[28]. However, the underlying mechanisms of gene body methylation in regulating gene expression have not been well understood. The causal relationships between gene body methylation and gene expression have been found in preventing transcriptional initiation from alternative promoters within genes, ensuring the accuracy of alternative-splicing and hindering transcriptional elongation [26], [29], [30]. Although an alternative hypothesis claimed that gene body methylation could be the by-product of expression [31], [32], the conservation of gene body methylation among orthologs in evolutionarily distant species and higher proportion of body-methylated genes with phenotypic effects supported that gene body methylation may play certain functional roles [33], [34].

Gene duplication plays a critical role in the origination of functional novelties in organisms. How duplicate genes evolve and become fixed in a genome is one of the central questions in molecular evolution [35]. Duplicate genes can be fixed through genetic drift or subfunctionalization/neofunctionalization driven by natural selection [35]–[45]. Previous studies revealed that the expression and function divergence of duplicate genes can be achieved through various mechanisms, such as nucleotide substitution, *cis*-regulation, post-translational regulation, promoter epigenetic marks and so on [37], [40], [46], [47]. A recent study demonstrated that the DNA methylation in promoter may play a significant role for functional divergence of duplicated genes in human [48].

In previous studies of methylation in duplicate genes, Wildman et al (2009) was the first to investigate the methylation pattern conservation between duplicate genes in *Arabidopsis* [49]. Chang and Liao (2012) discovered that DNA methylation in upstream flanking regions of genes repressed and thus “rebalance” the overall expression dosage of paralogs [46]. Wang et al (2013) found that ∼20% of the lineage-specific new duplicate genes had methylation pattern significantly more divergent from their parental genes in gene body than the methylation conservation of all the paralogs in *Arabidopsis* [50]. Wang et al. (2013) examined the divergence of gene body methylation levels of duplicate genes generated from different mechanisms, and the relationship between gene body methylation patterns and synonymous substitutions of paralogs [51]. However, it remains to be explored whether and how DNA methylation, particularly in gene body, influences the fate of duplicate genes in plants [52]–[54].

Here, we aim to determine how divergence of DNA methylation level and pattern in gene body is associated with expression divergence and evolution of duplicate genes in plant genomes. We chose rice (*Oryza sativa* ssp. *japonica*) and *Arabidopsis thaliana* as the model systems, which have high abundance of gene duplication and modest DNA methylation level in the genomes (in CG context, the methylation levels of rice and *Arabidopsis* are 23.27% and 38.21%, respectively, see Table S1 and [27], [55]). Our results show that gene body methylation divergence is associated with divergence of duplicate genes, suggesting that gene body methylation might play an important role in the expression divergence and evolutionary fate of duplicate genes in plant genomes.

## Materials and Methods

### Plant species selected and genome sequences, bisulfite-sequencing (BS-seq), expression and small RNA data sets

We selected two species, *A. thaliana*, and *O. sativa* ssp. *japonica* for our analyses. We downloaded their genome, CDS, protein sequences, and “.gff” files from Phytozome v8.0 http://www.phytozome.net/with *A. thaliana* 167 (TAIR10) for *A. thaliana*, and http://rice.plantbiology.msu.edu/downloads_gad.shtml (MSU7) for *O. sativa* ssp. *japonica*. We downloaded the BS-seq raw data from the NCBI Short Read Archive (SRA, http://www.ncbi.nlm.nih.gov/sra/) with accession numbers SRA000284 (immature floral tissue) for *Arabidopsis* [55] and SRA012190 (panicle) for *O. sativa* ssp. *japonica* [27], and from the NCBI Gene Expression Omnibus database (http://www.ncbi.nlm.nih.gov/geo/) with accession numbers GSM560562 (embryo), GSM560563 (endosperm), GSM560564 (seedling roots), and GSM560565 (seedling shoots) for rice [28], [56]. We downloaded the RNA-seq raw data from SRA with accession number SRA000286 (immature floral tissue) for *Arabidopsis* [55], and digital gene expression (DGE) raw data with accession number GSE20871 (panicle) from the NCBI Gene Expression Omnibus (GEO, http://www.ncbi.nlm.nih.gov/geo) for *O. sativa* ssp. *japonica* [27]. We downloaded *Arabidopsis* Affymetrix expression data of 56 microarray tissues/conditions that were not based on genetic mutants or overlapping tissues from http://www.ebi.ac.uk/arrayexpress/experiments/with accession number E-AFMX-9 [57]. We downloaded the RNA-seq data of endogenous small RNAs in panicle of rice from GSE32973 (GSM816731) of GEO [58], and that of small RNAs in wild-type immature flower of *Arabidopsis* from GSM277608 of GEO [55], which are the same types of tissues, where the methylation data were measured for the two species.

### Process methylation data, expression data and small RNA data

We re-analyzed the BS-seq data for *Arabidopsis* and *O. sativa* ssp. *japonica* with Bismark [59]. The intermediate steps included (1) running quality control and trimming the low quality bases with trim_galore (Version 0.2.5) (http://www.bioinformatics.babraham.ac.uk/projects/trim_galore/), (2) mapping the reads with Bismark v0.7.7, (3) removing the duplicates generated by PCR (deduplicate_bismark_alignment_output.pl), (4) generating cytosine methylation reports with Bismark v0.7.7. The plant chloroplast genome has no methylation activity [60], so any methylation reads detected in chloroplast genome should be accounted for the error. To test whether a cytosine is methylated, we conducted binomial test for each cytosine site based on the number of methylated reads, non-methylated reads and the error rate estimated from the chloroplast genome. To correct for multiple comparison problem, we computed the false discovery rate (FDR) ‘*q*’ value for each binomial test ‘*p*’ value [61]–[63]. The ‘*q*’ value≤0.05 was taken as the criterion of the methylated cytosine [27], [55]. We only considered the cytosine mapped with ≥5 BS-reads.

We reprocessed the raw RNA-seq data of *Arabidopsis* and DGE data of *O. sativa* ssp. *japonica*. For RNA-seq data, we trimmed the low quality data with trim_galore (Version 0.2.5), mapped the RNA-seq reads to *Arabidopsis* genome with Bowtie-0.12.8 [64], removed duplications due to PCR with picard-tools-1.79 (http://picard.sourceforge.net/), and finally we used cufflinks (v2.0.2) [65] to calculate gene-level relative abundance of reads in Fragments Per Kilobase per Million mapped fragments (FPKM) format. For reprocessing DGE data, we chose the longest transcript for each gene in *O. sativa* ssp. *japonica* genome to build the reference sequences, then mapped DGE reads to the reference sequences with Bowtie-0.12.8, finally counted the number of DGE reads for each gene and normalized the number of DGE reads with the gene length.

We annotated Affymatrix microarray expression data to *Arabidopsis* genes with customized CDF file downloaded from http://brainarray.mbni.med.umich.edu/Brainarray/Database/CustomCDF/17.1.0/tairg.asp. We processed the expression data with RMA package of R. The expression specificity index of each gene was computed according to the approach by Yanai et al [66]. Namely, we computed the tissue specificity index, τ.
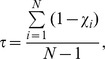
where N is the number of tissues, and 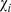 is the expression component (EC) in the *i*th tissue normalized by the maximal EC value among all the tissues. For the detailed steps to compute 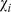 and τ, please refer to Yanai et al’s paper [66]. The τ value of each gene ranges from 0 to 1. The higher the τ value, the more specifically expressed the gene is [66].

We collected the processed counts of each small RNA in the genomes. We then mapped the small RNA sequences to the genomes of corresponding species with BWA (bwa-12–17–2013-git) [67]. Based on the above two information plus the coordinates of gene annotations, we estimated the number of small RNAs mapped in each paralogous gene.

### Identification and classification of paralogous gene pairs in the two species

We used all the peptide sequences of the genome to Blat [68] against all the peptides for the two species, respectively. We discarded the self-hit pairs and only kept the gene pairs whose reciprocal best hits were each other (namely, we only kept the gene pair of A and B, when the best hit of A is B and the best hit of B is A in the genome) with ≥70% sequence coverage and ≥50% sequence identity at the protein level. The Blat identity score was calculated following the formula in http://genome.ucsc.edu/FAQ/FAQblat.html#blat4. We classified the duplicate gene pairs according to four duplication mechanisms: dispersed duplication, retrotransposition, tandem duplication and segmental duplication. If a group (≥2) of genes, where any adjacent ones are within at most 10-gene distance, and their corresponding paralogs can also be clustered together with any adjacent ones within at most 10-gene distance, we defined them as generated by segmental duplication. If query and target genes of a paralogous pair were adjacent to each other, we defined them as generated by tandem duplication. If one duplicate gene had one exon, and the other copy had multiple exons, and intron losses in the CDS regions of the two genes were identified, we defined them as generated by RNA-based retrotransposition. The rest of paralogous gene pairs, both of which have single or multiple exons and are not belong to segmental, tandem duplication or retrotransposition, were defined as dispersed duplication. If a paralogous pair was classified into more than one duplication mechanism, we didn’t count this pair in the analyses of duplication mechanisms. We used Codeml of PAML4.7 [69] to compute Ka, Ks, and K for all the paralogs.

### Defining the methylation level/pattern divergence

For methylation level comparison, we only considered the paralogous pairs, where both genes have ≥50% of the cytosines mapped by BS-seq. We conducted Fisher test using 2×2 contingency table to compare numbers of methylated cytosines and non-methylated cytosines (in CG, CHG, CHH context, respectively) in the gene bodies of two paralogous genes. We used FDR (the ‘*q*’ value of the Fisher test ‘*p*’) to correct for multiple test problems. With the ‘*q*’ value of the Fisher test ‘*p*’ value≤0.05, we classified the paralogous pairs as the ones with divergent methylation level, otherwise as the ones with conserved methylation level. And we used the Fisher test ‘*p*’ value as the proxy of methylation level conservation to perform other analyses.

To define the paralogs with conserved methylation level in the promoter regions, we first chose different cutoff length of the regions at 5′ upstream transcription starting sites for two species respectively, and tested the correlation between methylation level and gene expression level in these regions. We identified that 200 bp in rice and 500 bp in *Arabidopsis* have the strongest correlation. Thus, we extracted 200 bp (for rice) and 500 bp (for *Arabidopsis*) upstream regions of 5′ end transcription starting sites as the potential promoter regions. We conducted Fisher test with 2×2 contingency table to compare the methylated cytosines and non-methylated cytosines in the promoter regions of the two paralogous genes. And we chose the paralogs with Fisher test ‘*p’* value and FDR ‘*q*’ value>0.05 as the ones with conserved methylation level in the promoter regions.

For methylation pattern comparison, we first aligned the CDS of the two paralogous genes with MAFFT [70]. We only considered the paralogous pairs with ≥50% of the aligned cytosines mapped by BS-seq. We classified the aligned cytosines in the same genomic context (both in CG, CHG, or CHH context) as conserved (both methylated or neither methylated), or non-conserved (one methylated and the other un-methylated). For each cytosine context, we computed the percentage of cytosines with conserved status, e.g., the number of cytosines with conserved status/(the number of cytosines with conserved +non-conserved status), as the proxy of methylation pattern conservation for other analyses. We then computed the average percentage of methylation pattern conservation for all the paralogs. We conducted a binominal test with this average percentage, the number of cytosines with conserved status, and the number of cytosine with non-conserved status. We computed the FDR ‘*q*’ value associated with the ‘*p*’ value of binominal test. We defined the paralogs with pattern conservation percentage significantly (FDR ‘*q*’ ≤0.05) lower than the average value as the ones with non-conserved methylation patterns, and the rest as those with conserved methylation patterns.

We further divided paralogous gene pairs into conserved highly methylated, conserved lowly methylated, and conserved non-methylated pairs. If the methylation of both paralogous copies was zero, we classified the pair into the conserved non-methylated group. If the methylation levels of both copies of a pair were higher than the median methylation level of all paralogs with conserved methylation level/pattern, we classified this pair into the conserved highly methylated group. And if they were lower than the median, we classified the pair into the conserved lowly methylated group.

### Population genetics analysis and mutation frequency estimation

We obtained *A. thaliana* single nucleotide polymorphism (SNP) data generated from a complete re-sequencing of 80 strains using next-generation sequencing technology [71], downloading from http://1001genomes.org/data/MPI/MPICao2010/releases/current/. We then computed the 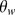 values for duplicate genes in *Arabidopsis*. 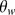 was defined as the number of segregation sites, which can be estimated using SNP data, divided by 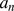 where 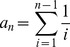 [72].

To estimate the G/C to A/T mutation frequency of *A. thaliana* genes, we compared *A. thaliana* gene to its *A. lyrata* orthologous sequences. The extracted genomic sequences of *A. thaliana* genes were Blat against *A. lyrata* genome. The genomic sequences of *A. thaliana* genes and their *A. lyrata* orthologous sequences were identified and then aligned. We collected the SNPs of the aligned regions of these *A. thaliana* genes. For each SNP, the derived allele was distinguished from ancestral using the *A. lyrata* orthologous sequence as ougroup. We only considered the G/C→A/T mutations in the sites of the *A. thaliana* gene. The mutation frequency of G/C→A/T was calculated from the quotient of the total number of G/C→A/T mutations and the total number of G/C bases in the aligned region of *A. thaliana* genes.

### Analysis of TE distribution of paralogs

We downloaded the repeat library repeatmaskerlibraries-20130422 from http://www.girinst.org/, and mapped the repeat sequences to the genome with RepeatMasker4.0.1 [73]. Based on the mapping result, we divided paralog pairs into three categories: 1) both copies contain TEs/repeat sequences within 1 kb upstream or downstream region; 2) neither copy has TEs/repeat sequences within 1 kb upstream or downstream region; 3) only one of the two copies has TEs/repeat sequences within 1 kb upstream or downstream region. We conducted further analyses based on this classification. Additionally, we used different cutoff length of paralog flanking region to search for the nearby TEs and examined the reliability of our analyses.

### Statistical analyses

Correlations were measured with Spearman’s rank correlation method. We used ‘ppcor’ package in R to compute partial correlation [51], [74], [75]. ‘ppcor’ can calculate the pairwise partial correlations for each pair of variables while controlling a third or more other variables with three correlation methods (i.g. Pearson, Kendall, and Spearman). Each variable has mathematical expressions and variances to compute the partial correlation with the response variable [76]. It provides the ‘*p*’ value as well as statistic for each pair of variables.

The goodness of fit was measured with *R*-squared (coefficient of determination, *R^2^*), and coefficient significance of linear regression with ‘lm’ function in R. *R^2^* is defined as the percentage of the response variable variation that can be explained by a statistical model, so *R^2^* =  explained variation/total variation, and specifically 
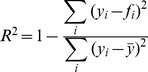
. The binomial test of the significance of paralog pairs overlapped in the same categories of the two species was conducted using ‘binom.test’ function in R. The expected probability to overlap is P = (conserved speciesA%*conserved speciesB%+non-conserved speciesA%*non-conserved speciesB%). The number of paralog pairs with data available in both species A and species B is ‘N’, and the observed number of paralog pairs with same categories in the two species is ‘X’.

All the intermediate steps were carried out with Perl and R scripts.

## Results

### Plant duplicate genes identification and gene body DNA methylation measurement

**Figure 1:**
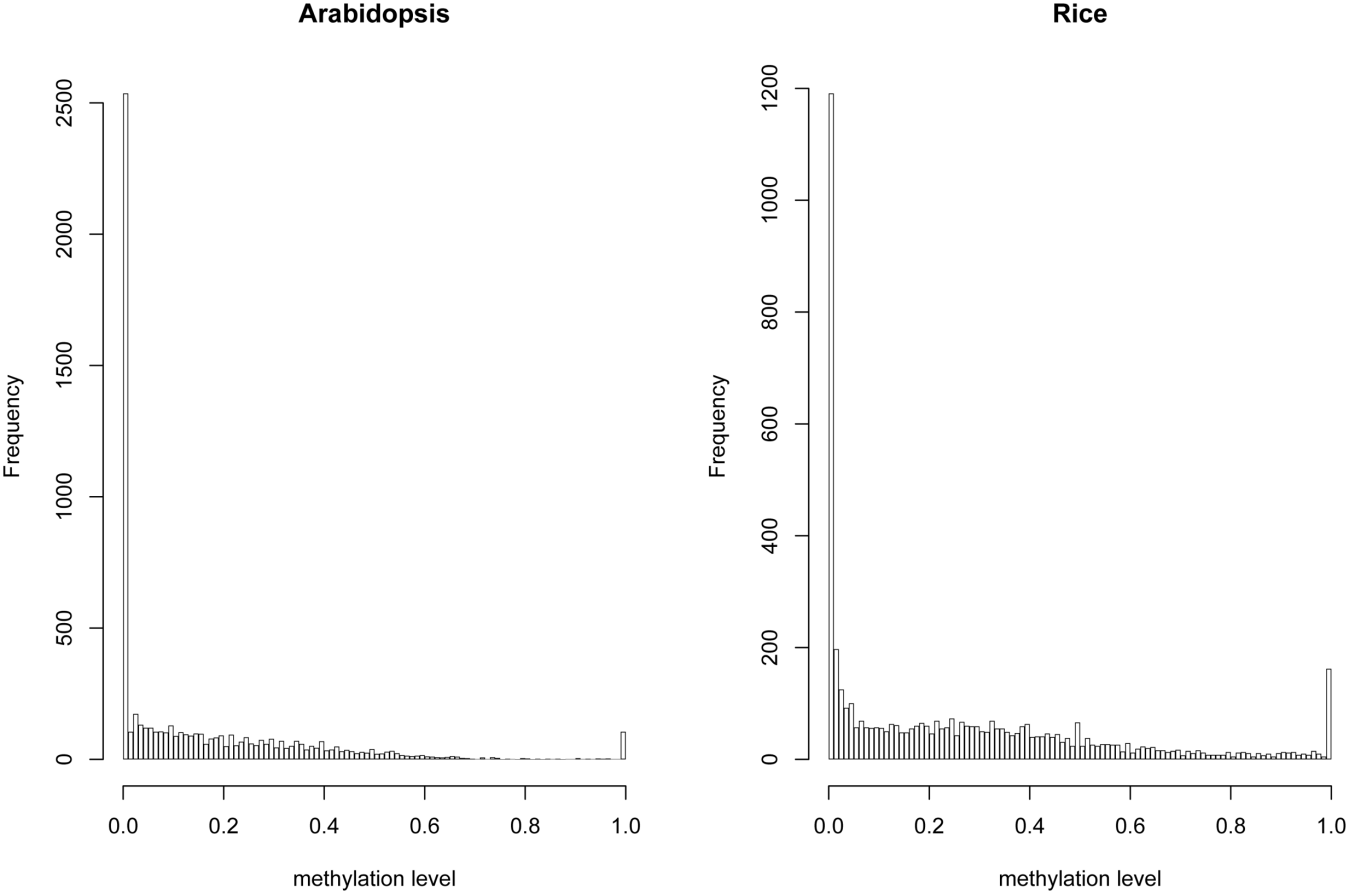
Histograms of methylation level of all duplicate genes in *Arabidopsis* and rice.

We identified total 3459 and 2911 paralogous gene pairs in *Arabidopsis* and rice genomes, respectively. We first reprocessed the BS-seq data generated from the immature floral tissue of *Arabidopsis* and the panicle of rice. Our reprocessed results are consistent with previous reports (Table S1) [27], [55]. We assessed the DNA methylation divergence between the two paralogous genes from two aspects: (1) methylation level, which estimates the overall methylation level difference in the entire gene body; (2) methylation pattern, which estimates the effect of methylation change in specific cytosine sites. The methylation level was defined as the percentage of methylated cytosines over the total cytosines mapped by BS-seq, for each of the cytosine contexts respectively. Methylation pattern was measured by the percentage of cytosines with conserved methylation status (both methylated/neither methylated) among all mapped aligned cytosines in the coding sequences (CDS) of paralogs, for each of the cytosine contexts respectively. Gene body is typically only methylated in the CG context, so methylation on cytosine in CG context is most abundant in gene body [55], [77] and CG methylation in genic region is associated with up-regulation of gene expression [78]. Whereas, CHG and CHH methylation is often associated with RNA-directed DNA methylation and represses gene transcription [5]. Therefore, we focused on CG methylation in the following analyses. Our results showed that the methylation levels of all duplicate genes are bi-modally distributed for both species (Figure 1).

**Figure 2:**
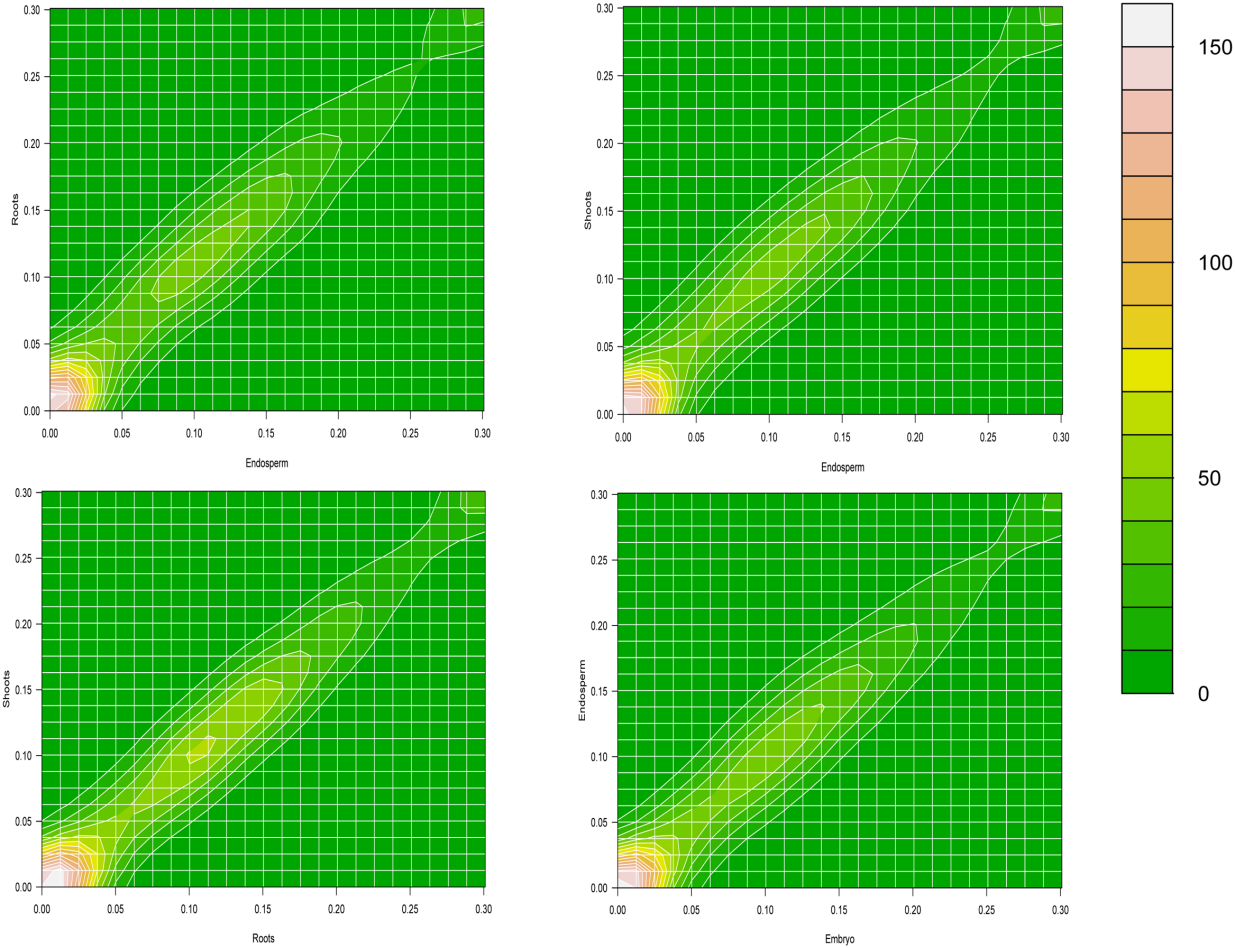
Contour plots of gene body methylation level of rice duplicate genes among embryos, endosperms, seedling roots, and seedling shoots. The ‘x’ axis and ‘y’ axis are methylation level in two tissues, respectively. We take log(methylation level +1, base = 10) as the measured values, since a large portion of genes with methylation level = 0.

Further, to examine the consistency of gene body methylation level of duplicate genes across different tissues, we reprocessed the raw BS-seq data from embryos, endosperms, seedling roots, and seedling shoots in rice. We pair-wisely plotted the gene body methylation levels of rice duplicate genes among the four tissues with the contour plots. The methylation levels between any pair of the tissues are highly correlated (Spearman’s rank correlation coefficients of all pairs of tissues >0.96, *p*<2.2e-16, Figure 2). This result suggested that the majority of duplicate genes have similar methylation levels across different tissues with few genes with tissue-specific methylation pattern, which is consistent with previous results for all the genes [34], [51]. Therefore, it is rational to use the methylation level from one tissue (e.g. the immature floral tissue of *Arabidopsis* and the panicle of rice) as the representative to study the general patterns of methylation for a large set of genes in the following analyses.

### Gene body DNA methylation correlates with gene properties

To investigate whether the methylation divergence influences the divergence of duplicate genes, we first measured the relationship between DNA methylation and the properties of duplicate genes including gene structure, transcription and mutation rate.

#### DNA methylation and gene structure

**Figure 3:**
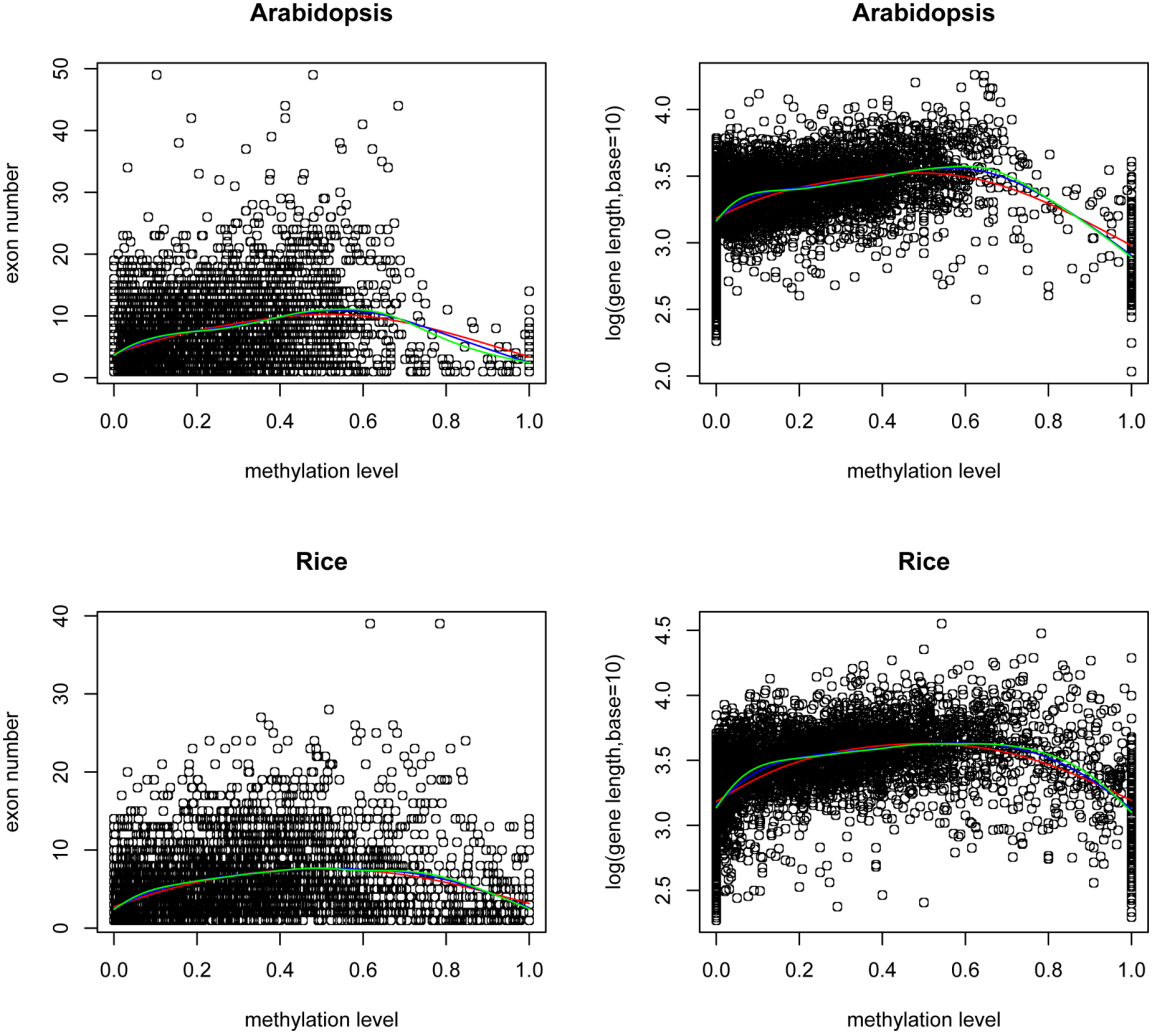
Plots of the methylation level *vs.* gene length/exon number for duplicate genes in *Arabidopsis* and rice. The red, blue, and green lines are corresponded to smoothing spline regression from degree of freedom (*df*) as 4, 6 and 8, respectively. The different *df* values were taken to avoid the over fitting in smoothing spline regression. 0.6 appears as the inflection point of the curves.

Previous studies revealed inconsistent relationships between gene length/exon number and methylation. For example, in honeybee and silkworm, lowly methylated genes were significantly longer than highly methylated ones; whereas in the sea squirt and sea anemone, highly methylated genes were significantly longer than lowly methylated ones [79]. And in *Arabidopsis* body-methylated genes are longer and have more exons [33], [80]. Using smoothing spline function in R with different degrees of freedom, we plotted the regression line between the methylation level and gene length/exon number for duplicate genes in *Arabidopsis* and rice, respectively [51]. The methylation level 0.6 was present as the inflection point dividing the positive and negative correlation with gene length/exon number (Figure 3). Thus we investigated this relationship by separating the duplicate genes into two groups with methylation level <0.6 and ≥0.6. We found that methylation level positively and negatively correlates to gene length/exon number for the two groups of genes, respectively. Total number of duplicate genes in *Arabidopsis* and rice are 6498 and 4992, respectively. Spearman’s rank correlation *p*<2.2e-16 and *R^2^* of linear regression model in the two methylation intervals are in the range of 0.14–0.38. The linear coefficients are statistically significant with *p* value<2e-16 (Table S2). However, the data size of duplicate genes with methylation level ≥0.6 was small with 247 in *Arabidopsis* and 631 in rice, and 26%–43% of them fell into the range of methylation level = 1 (Figure 3), which is corresponding to the methylation level distribution of duplicate genes (Figure 1). Therefore, given the biased sampling, it needs caution to interpret the correlation in this interval. Further, to explore the relative association of gene length and exon numbers with methylation level and their interactions, we examined the partial correlation among the methylation level, gene length and exon number with the “ppcor” package in R. We found gene length and exon number are highly correlated with Spearman’s rank correlation coefficient around 0.7 and *p*≈0. Gene length is more closely correlated to methylation level than exon number.

Our results show that the gene length/exon number does not linearly correlate to methylation level and intermediately methylated genes are longer and contain more exons in plants. The underlying mechanisms of gene body methylation remain to be explored. However, our analyses imply that gene body methylation may prevent transcriptional initiation from cryptic promoters within genes. Therefore, longer genes with more exons require higher methylation level to ensure their precise expression. Thus methylation level is positively correlated with gene length/exon number below certain threshold. Meanwhile, extreme high methylation level could hinder transcriptional elongation, so shorter genes can be associated with higher methylation level as well. Thus methylation level is negative correlated with gene length/exon number after gene body methylation level passes certain threshold.

#### DNA methylation and gene expression

**Figure 4:**
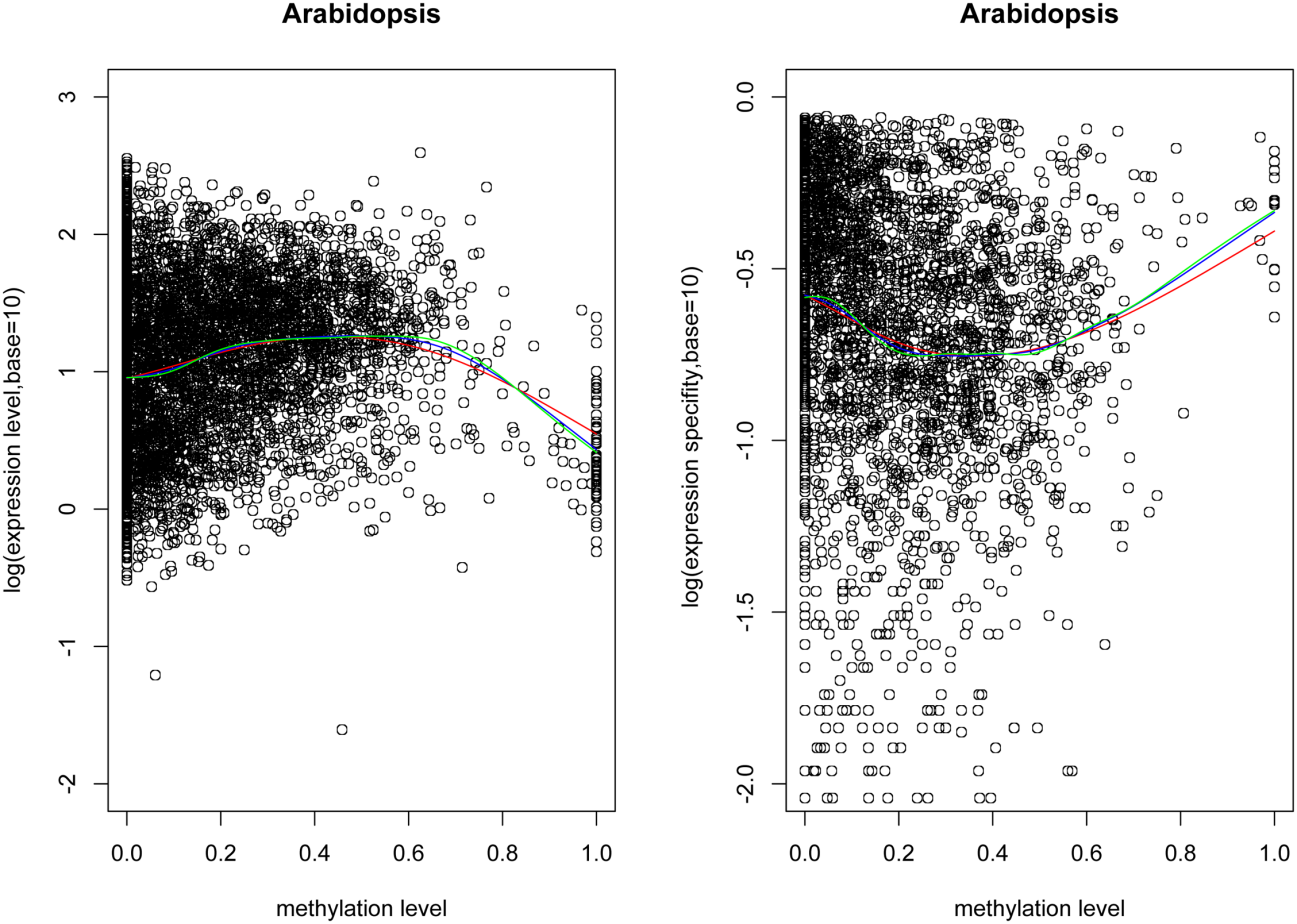
Plots of methylation level *vs.* expression level/specificity for duplicate genes in *Arabidopsis*. The red, blue, and green lines are corresponded to smoothing spline regression from degree of freedom as 4, 6 and 8, respectively. 0.5 appears as the inflection point of the curves.

We measured the correlation between gene body methylation and gene expression in terms of expression level and specificity in *Arabidopsis*. Expression level was measured with RNA-seq in the immature floral tissue where the methylation was measured. Whereas, the expression specificity was computed based on 56 tissues/conditions. Previous studies showed that moderately methylated genes had higher expression level, and extremely high or low methylation accompanied lower gene expression [27]. We plotted correlation between methylation level and gene expression level/specificity. Methylation level 0.5 appears as the turning point of the curves, which was determined based on smoothing spline regression (Figure 4). Therefore, we separated the duplicate genes into two groups with methylation level <0.5 and ≥0.5. For the ones with methylation level <0.5, expression level positively and expression specificity negatively correlate with methylation level; and for the ones with methylation level ≥0.5, expression level negatively and expression specificity positively correlate with methylation level. Spearman’s rank correlation *p*<0.05 and *R^2^* of linear regression model in the two methylation intervals are in the range of 0.0079–0.2873 (Table S3). The linear coefficients are statistically significant with *p* value<1e-5 (Table S3). Additionally, the expression level and specificity strongly negatively correlate with each other (Spearman’s rank correlation coefficient: −0.6845 p<2.2e-16).

The small *R^2^* between gene body methylation and expression may be due to regulation of gene expression by multiple factors, so the contribution of gene body methylation to expression is relatively small and/or mirrored by other confounding factors. Thus, we examined the general correlation between expression levels/specificity and other factors including small RNA abundance (we focused on the 21nt and 24nt small RNA here, see explanation in the later section), promoter methylation, gene length, exon number, and mutation rate. Mutation rate was measured by theta (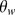) estimated from SNPs [81]. We found that expression level was significantly negatively associated with the 21nt and 24nt small RNA abundance, promoter methylation level, and mutation rate (*p*<2.2e-16), but was positively associated with gene length and exon number (*p*<2.2e-16, Table S4). Expression specificity was significantly negatively associated with gene length and exon number (*p*<2.2e-16), but was positively associated with 21nt and 24nt small RNA abundance, promoter methylation level, and mutation rate (*p*<1e-7, Table S4).

To control for the effects of the above factors and test whether gene body methylation exclusively correlates to gene expression, we computed the partial correlation between methylation level and expression level/specificity, considering all the above factors simultaneously using the “ppcor” package of R [74], [75]. Our analyses show that the partial correlations between methylation and expression are similar but weaker than the above general correlations (Spearman’s rank correlation coefficients. Methylation level <0.5: *vs.* expression level: 0.1375, *p* = 6.760e-16; *vs.* expression specificity: −0.1694, *p* = 3.9333e-17. Methylation level ≥0.5: *vs.* expression level: −0.0898, *p* = 0.1102; *vs.* expression specificity: 0.0934, *p* = 0.1858). These results suggest that gene body methylation is indeed associated with gene expression, and the associations for duplicate genes are consistent with the overall association for the non-TE genes revealed by the previous studies [25]–[28], [33], [77].

#### DNA methylation and gene mutation rate

**Figure 5:**
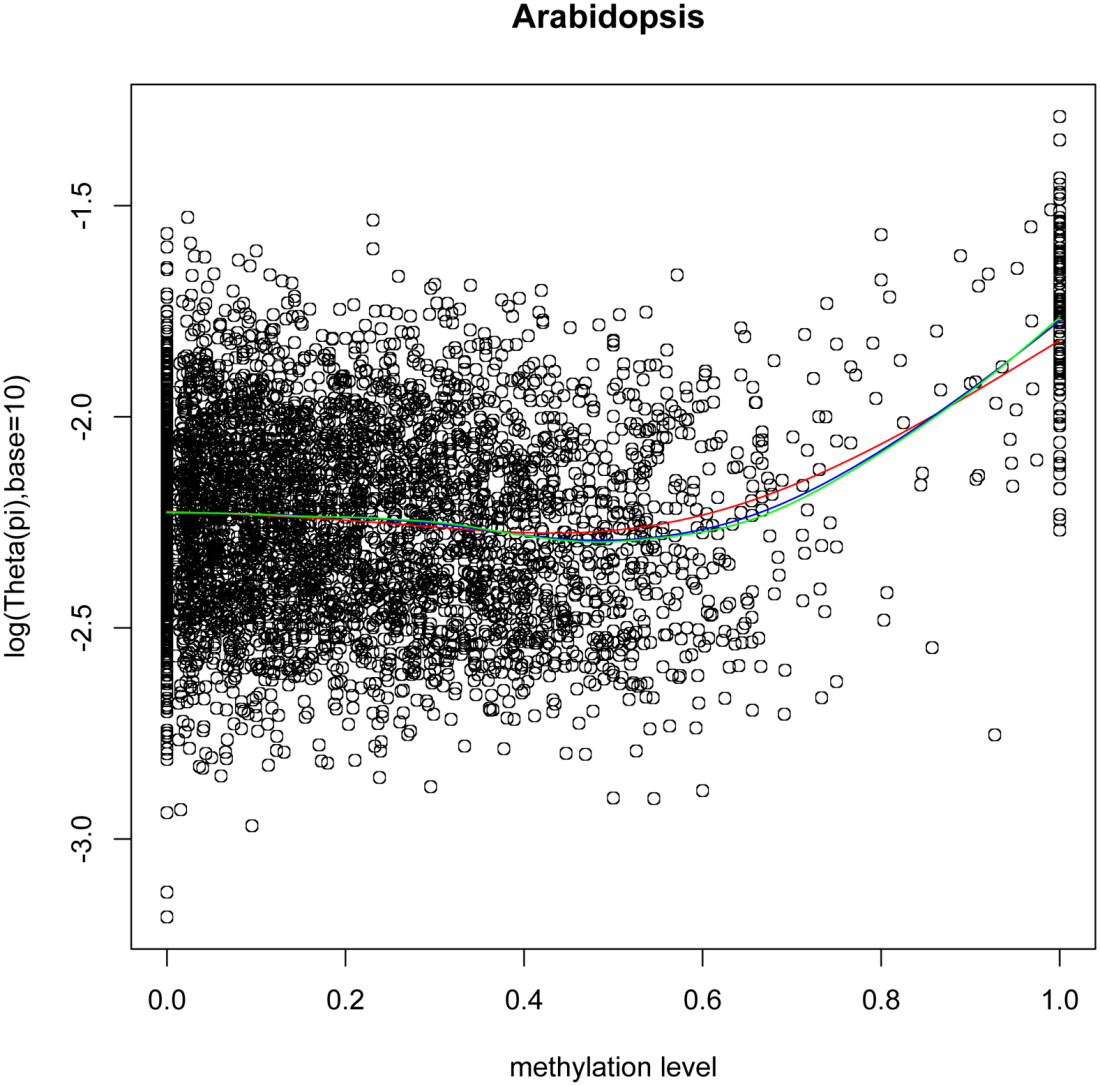
The plot of methylation level *vs.* mutation rate 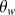 for duplicate genes in *Arabidopsis*. The red, blue, and green lines are corresponded to smoothing spline regression from degree of freedom as 4, 6 and 8, respectively.

To investigate the association between methylation level and sequence changes of duplicate genes, we looked into the correlation between methylation level and DNA sequence mutation rate, 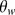, in *Arabidopsis* as the SNP data corresponding to the current reference genome was only available in *Arabidopsis*. Given the inflection point of the regression line between 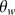 and methylation level as 0.5 (Figure 5), we also split the duplicate genes into two groups with methylation level <0.5 and ≥0.5. Methylation level negatively and positively correlates with 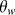 for the two groups of genes, respectively. Spearman’s rank correlation *p*<1e-7 and R^2^ of linear regression model in the two methylation intervals are in the range of 0.0070–0.4304. The linear coefficients are statistically significant, *p* value<1e-7 (Table S5). However, the correlation of 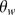 and other factors including exon number, gene length, expression, small RNAs and promoter methylation level could be the cofounding factors between methylation level and 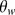 (Table S6). After controlled all these factors simultaneously, we still observed the consistent partial correlations between 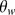 and methylation level (Spearman’s rank correlation coefficients: Methylation level <0.5: 0.0004, *p* = 9.7994e-01. Methylation level ≥0.5: 0.2241, *p* = 4.6118e-05). This result suggests that highly methylated genes have higher mutation rates and intermediately methylated genes have lower mutation rates. This is conceivable, since highly methylated genes may experience higher mutation rate due to the mutagenic property of the methylated cytosines, which frequently mutate to thymine [82]–[84]. To further test this speculation, we computed frequency of G/C→A/T mutation as the percentage of G/C sites with G/C→A/T mutation among all the G/C sites for *Arabidopsis* genes based on *Arabidopsis* SNP data and *A. lyrata* genome sequence (see Method and Materials). We found that when methylation level <0.5, G/C→A/T mutation frequency is negatively correlated with methylation level (Spearman rank’s correlation coefficient = −0.1853, *p*<2.2e-16). Whereas, G/C→A/T mutation frequency is positively correlated with methylation level when methylation level ≥0.5 (Spearman rank’s correlation coefficient = 0.1100, *p* = 0.01869). Thus, this result supported the above conjecture, suggesting methylation and DNA mutation may be able to interact with each other.

### Methylation divergence positively correlates with genetic divergence of paralogs

We estimated how divergence of methylation level/pattern was associated with genetic divergence of paralogs, measured by synonymous (Ks) and non-synonymous (Ka) substitution rate. Previous studies suggested that orthologous genes with high methylation levels in both species evolved more slowly than those with low methylation levels in both species, or those with high methylation level in one species but low methylation level in the other species [33], [79]. Wang et al (2013) found that the methylation level divergence of paralogs was positively correlated with Ks given the gene physical distance (measured by the number of genes between two paralogs) and duplication mechanisms [51]. Consistent to previous study [51], we observed that the physical distance of paralogs negatively correlated with the conservation of methylation level and pattern (Spearman’s rank correlation coefficients. *Arabidopsis* methylation level: −0.0582, *p* = 4.375e-03 and methylation pattern: −0.1068, *p* = 1.937e-07. Rice methylation level: −0.0217, *p* = 0.3082 and methylation pattern: −0.0848, *p* = 7.708e-05). Further, we found that the physical distance positively correlated with Ks or Ka (Spearman’s rank correlation coefficients. *Arabidopsis* Ks: 0.4639, *p*<2.2e-16 and Ka: 0.0586, *p* = 4.076e-4. Rice Ks: 0.2799, *p*<2.2e-16 and Ka: −0.0033, *p* = 0.8775).

Physical distance could be a confounding factor for the relationship between Ks or Ka and methylation conservation. Therefore, we computed the partial correlation between Ks or Ka and methylation level/pattern conservation given physical distance. We found that methylation level conservation negatively correlates with both Ks and Ka in both genomes, but methylation pattern conservation only negatively correlates with Ks and Ka in rice (Spearman’s rank correlation coefficients, *p*<0.05, Table 1; also see Figure S1). Theoretically, Ks implies the divergent time or neutral DNA evolutionary rate, and Ka suggests the amino acid changes. Thus, these results suggest that methylation level divergence of paralogs may increase along with evolutionary time and correlate with the DNA and protein sequence divergence. For example, genetic variables could give rise to methylation divergence or *vice versa* as we showed the correlation between methylation level and mutation rate. Further, no significant correlations between methylation pattern divergence and Ks in *Arabidopsis* could be explained by the process of recurrent methylation and demethylation at specific sites/regions in short period [85].

**Table 1:** The partial Spearman’s rank correlations between methylation level/pattern conservation and substitution rates in *Arabidopsis* and rice.

Rice	Ks∼level	Ka∼level	Ks∼pattern	Ka∼pattern
Coefficient	−0.2218	−0.2175	−0.1175	−0.0702
*p* value	1.242e-26	1.241e-25	3.753e-08	0.0011
***Arabidopsis***	**Ks∼level**	**Ka∼level**	**Ks∼pattern**	**Ka∼pattern**
Coefficient	−0.1644	−0.1108	−0.0230	0.0212
*p* value	3.386e-16	4.872e-08	0.2634	0.3022

Note: Correlation coefficient and *p* value were computed with Spearman’s rank sum correlation.

### Methylation divergence is associated with the expression divergence of paralogs

As shown by the above analyses that gene body methylation indeed correlated with gene expression, we examined whether divergence of gene body methylation contributes to expression divergence of paralogs. We divided paralogs into four categories based on the conservation of methylation level/pattern in gene body: conserved highly methylated (CHM), conserved lowly methylated (CLM), conserved non-methylated (CNM), and non-conserved methylated (NCM) (see Materials and Methods). The numbers of paralogous pairs in the four categories for the two species were listed in Table 2.

**Table 2:** The number of paralogous genes with different gene body methylation conservation.

Paralogs	For methylation level		For methylation pattern	
	*Arabidopsis*	Rice	*Arabidopsis*	Rice
CHM	633	403	565	463
CLM	627	402	558	462
CNM	758	159	737	156
NCM	797	729	561	456

Note: CHM-Conserved Highly Methylated; CLM-Conserved Lowly Methylated; CNM- Conserved Non-Methylated; NCM- Non-Conserved Methylated.

We further test whether the patterns of methylation divergence of duplicate genes were consistent across different tissues in rice. Interestingly, for methylation level divergence, most duplicate gene pairs (85.16%–89.50%) were classified into the same categories (either conserved or non-conserved) for any pair of the tissues (e.g. embryos, endosperms, seedling roots, and seedling shoots). For methylation pattern, similarly, the majority of duplicate gene pairs (88.22%–92.63%) were in the same categories for any pair of the tissues. Binomial tests indicate that the proportion of these observed gene pairs in either methylation level or pattern are significantly larger than random expectation (*p* value<2.2e-16). This result suggested that duplicate genes in general maintained the same patterns of methylation divergence across different tissues, which is consistent with the observation in human tissues [48] and was first reported in plants here. It also implies that it is feasible to use the methylation divergence of duplicate genes in one tissue to study the general relationship between methylation divergence and expression divergence.

**Figure 6:**
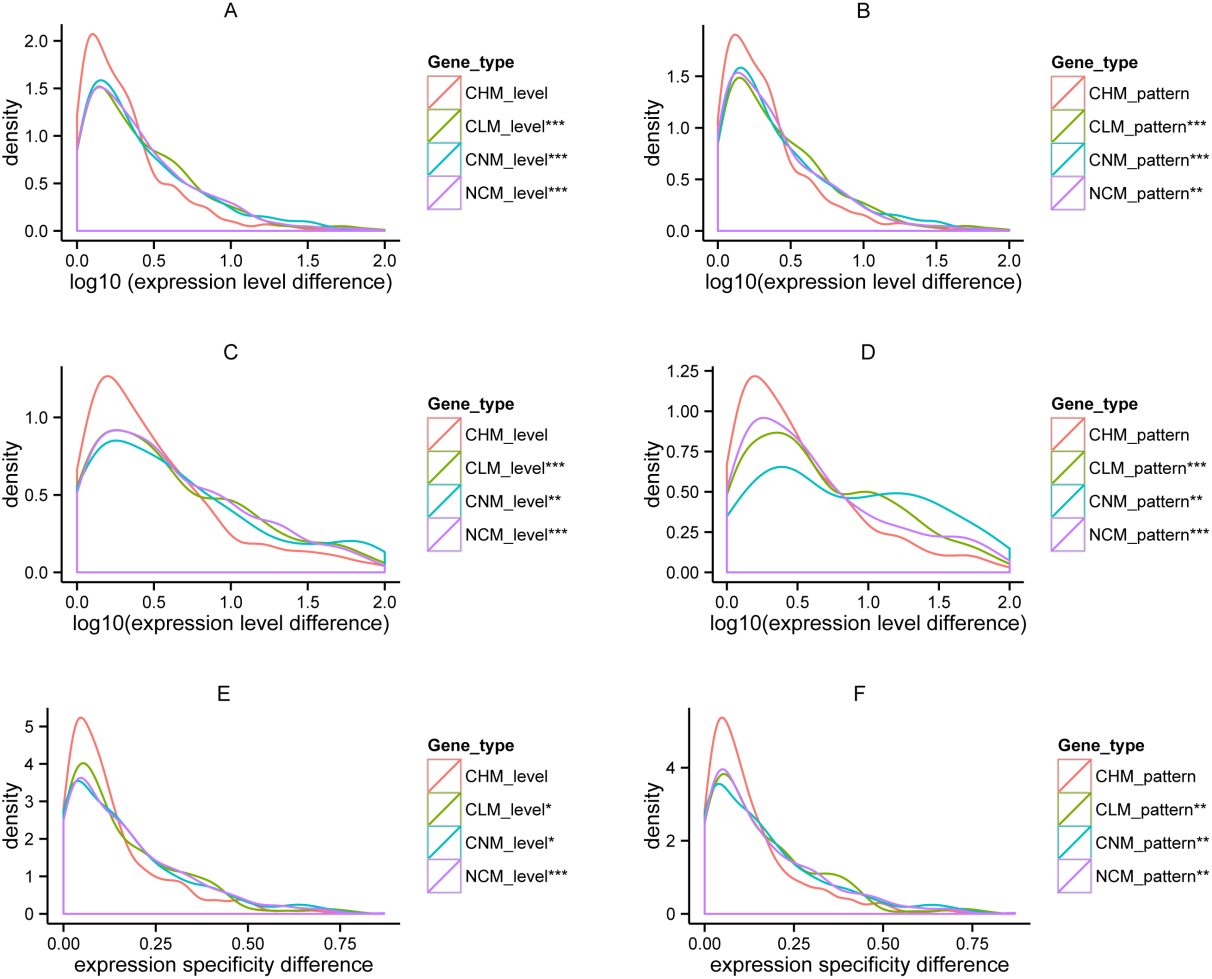
Relationship between expression and methylation divergence of paralogs in *Arabidopsis* and rice. A: Distribution of expression **fold** changes of *Arabidopsis* paralogs in the three methylation **level** conservation categories. B: Distribution of expression **fold** changes of *Arabidopsis* paralogs in the three methylation **pattern** conservation categories. C: Distribution of expression **fold** changes of rice paralogs in the three methylation **level** conservation categories. D: Distribution of expression **fold** changes of rice paralogs in the three methylation **pattern** conservation categories. E: Distribution of expression **specificity** changes of *Arabidopsis* paralogs in the three methylation **level** conservation categories. F: Distribution of expression **specificity** changes of *Arabidopsis* paralogs in the three methylation **pattern** conservation categories. CHM: conserved highly methylated paralogs. CLM: conserved lowly methylated paralogs. CNM: conserved non-methylated paralogs. NCM: non-conserved methylated paralogs. Significant levels (*) are assigned to CLM, CNM, or NCM If their expression changes are significantly higher than those of CHM by Wilcoxon rank sum test. Significant levels were shown as “*”: *p*<0.05, “**”: *p*<0.01, and “***”: *p*<0.001.

We computed the expression level fold change and expression specificity difference between the two paralogous genes as the two proxies of their expression divergence, and compared the two proxies among the four categories. The expression level fold change was determined by the absolute quotient of expression level of two paralogous genes and the paralog with higher expression level was always assigned as numerator. We found the expression level and specificity changes of CHM paralogs were significantly lower than those of CLM, CNM and NCM paralogs for the two species in both methylation level and pattern (Figure 6. Wilcoxon rank sum test, *p*<0.05, the detailed *p* values see Table S7 and sample size see Table 2).

### Cofounding factors that associated with methylation and expression divergence of paralogs

As shown previously, expression level/specificity could be affected by other factors, e.g. nucleotide substitution, difference in gene structure (gene length and exon number), *cis*-regulatory motifs, promoter methylation, small RNA abundance, and physical distance (Table S4). Thus expression divergence of paralogs could also result from these factors. We controlled these factors one by one and finally altogether, to determine whether and how the methylation divergence was associated with expression divergence of duplicate genes. Due to the similar behavior of CNM and CLM (Figure 6) and CNM paralogs may be not regulated by methylations, we only performed the comparison among CHM, CLM, and NCM in the following analyses.

#### The effect of gene structure

To control for the effect of gene structure difference, we choose the duplicate genes with the same ranges of exon number and gene length, which are not significantly different among the three categories in *Arabidopsis* and rice. We found the expression level changes of the CHM paralogs are significantly lower than those of the CLM and NCM ones for methylation level and pattern in both genomes (Wilcoxon rank sum test, *p*<0.05, Table S8).

#### The effect of nucleotide substitutions

**Figure 7:**
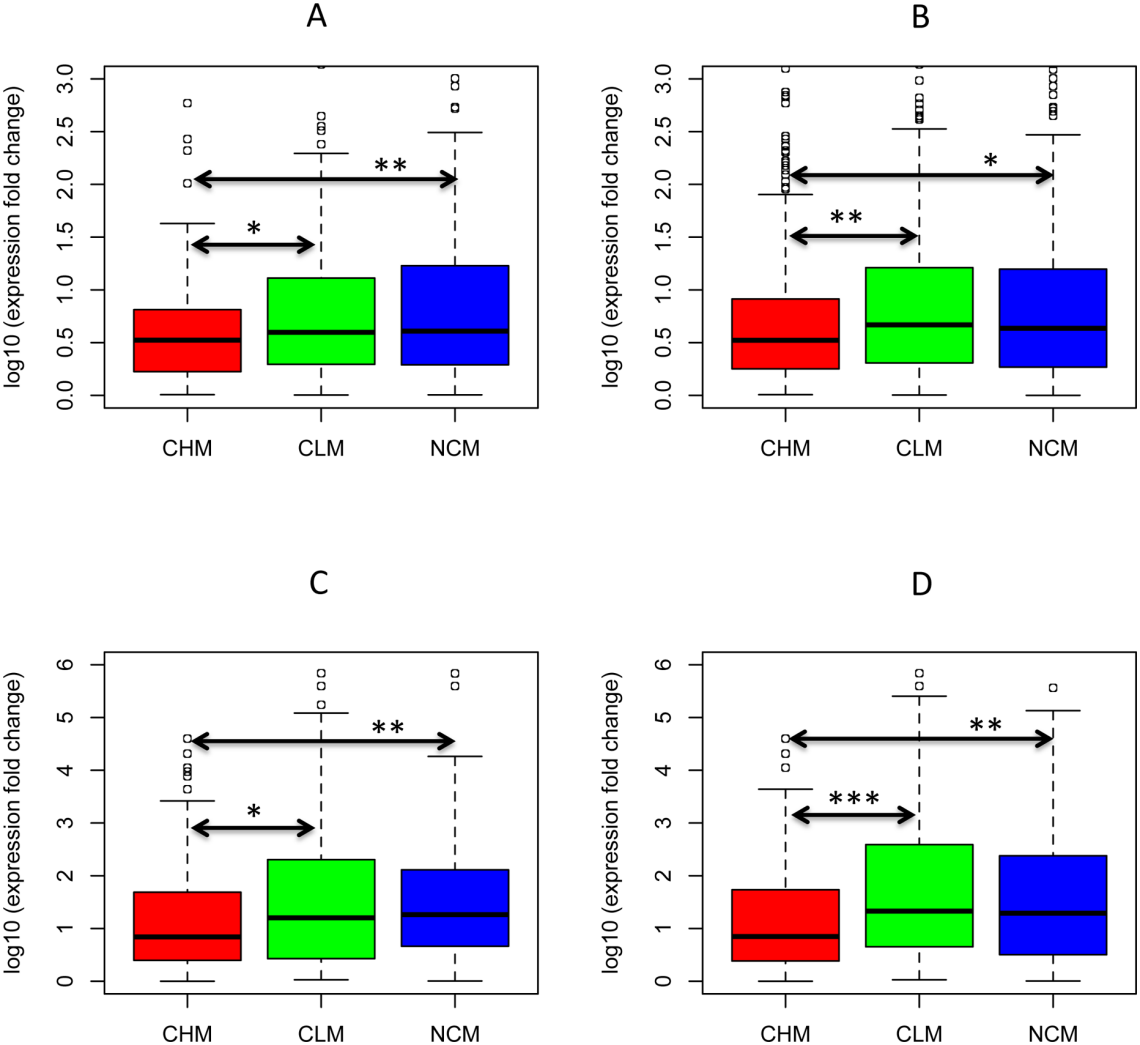
Relationship between methylation level and expression divergence of paralogs in *Arabidopsis* and rice genomes controlling DNA substitution rates (K) in the certain range, which are not significant different among the three methylation conservation categories. A: boxplot of expression **fold** changes of *Arabidopsis* paralogs in the three methylation **level** conservation categories with K in the range of 0.05–0.2. B: boxplot of expression **fold** changes of *Arabidopsis* paralogs in the three methylation **pattern** conservation categories with K in the range of 0.01–0.3. C: boxplot of expression **fold** changes of rice paralogs in the three methylation **level** conservation categories with K in the range of 0.05–0.25. D: boxplot of expression **fold** changes of rice paralogs in the three methylation **pattern** conservation categories with K in the range of 0.05–0.25. Significant levels of Wilcoxon rand sum test were shown as “*”: *p*<0.05, “**”: *p*<0.01, and “***”: *p*<0.001.

To control for the effects of nucleotide substitutions, we calculated the nucleotide substitution rate (K) in CDS regions, for each of the paralogs, in *Arabidopsis* and rice. We choose the paralogs with the same range of K values (e.g. in the range of 0.05 to 0.2), which were not significantly different among the three categories (Table S9). We found the expression level differences/specificity differences of the CHM duplicate genes are significantly lower than those of the CLM and NCM ones in methylation level and pattern for both species (Figure 7). Wilcoxon rank sum test shows *p*<0.05 except the comparison of expression specificity differences between CHM and CLM for methylation level (*p* = 0.05418), and between CHM and CLM for methylation pattern (*p* = 0.05397) (Table S9).

#### The effect of cis-regulatory difference

To control for the effect of *cis*-regulatory difference, we extracted the *cis*-regulatory binding sites of *Arabidopsis* duplicate genes, based on all the *cis*-regulatory binding sites in *Arabidopsis* genome downloaded from AGRIS (http://arabidopsis.med.ohio-state.edu/downloads.html). We picked the paralogous pairs whose two copies have the same types of *cis*-regulatory binding sites, we still observed that the expression level divergence of the CHM paralogs is significantly lower than that of the NCM ones in methylation level (Wilcoxon rank sum test, *p* = 0.0565 with 101 CHM paralogs and 103 NCM paralogs). However, because the false positive rate of *cis*-regulatory binding site mapping is high, this analysis might not be able to completely and precisely control the effect of *cis*-regulatory difference.

#### The effect of promoter methylation difference

To control for the effect of promoter methylation difference, we choose the paralogous pairs with conserved methylation level (using Fisher test, see Materials and Methods) in their promoter regions in *Arabidopsis* and rice. Among them, we found that the expression level/specificity changes of the gene body CHM paralogs were significantly lower than those of the CLM and NCM ones in methylation level and pattern for both genomes (Wilcoxon rank sum test, *p*<0.05, except the comparison of expression specificity changes between CHM and CLM for methylation level *p* = 0.1246. Table 3).

**Table 3:** Comparison of expression divergences (*p* value) in three gene body methylation conservation categories after controlling promoter methylation divergence.

Species	Expression level divergence		Expression specificity divergence	
*Arabidopsis*	CHM<CLM	CHM<NCM	CHM<CLM	CHM<NCM
Methylation level	3.432e-06	7.388e-07	0.1246	0.02
Methylation pattern	4.929e-04	0.02239	0.005676	0.005267
**Rice**	**CHM<CLM**	**CHM<NCM**		
Methylation level	1.831e-05	1.458e-05		
Methylation pattern	2.067e-07	8.958e-04		

Note: *p* value was computed with Wilcoxon rank sum test.

#### The effect of post-transcription regulation

To control for the effect of post-transcriptional regulation (e.g. regulation of the small RNAs), we mapped small RNA sequences to *Arabidopsis* and rice genomes and estimated the numbers of the small RNAs mapped to each duplicate gene normalized by gene length. Because small RNAs with different length may regulate gene expression differently [86]. To simplify and standardize the experiment, we focused on 21nt and 24nt small RNAs, which are the most abundant small RNA species in *Arabidopsis* and rice [55], [58], [87] and can influence gene expression through microRNA and siRNA induced DNA methylation and microRNA mediated mRNA cleavage [8], [86], [88]. Then, we choose gene pairs with similar proportions (the number of small RNAs/the gene length) of 21nt and 24nt small RNAs respectively between the two paralogous copies, and found that the expression level changes of the CHM paralogs were still significantly lower than those of the CLM and NCM ones for most comparisons in methylation level and pattern for the two genomes (Wilcoxon rank sum test, *p*<0.05, although some comparison had insignificant *p* values (Table S10). However, the results should be interpreted with the caution since small RNAs data used here were limited and our operation may not completely control the effect of post-transcription regulation.

#### Controlling all factors simultaneously

Finally, to control for nucleotide substitution, differences in exon number/gene length, promoter methylation and small RNA abundance, and physical distance simultaneously, we pooled the CHM and NCM duplicate genes together to compute the partial correlation between expression divergence and methylation conservation while controlling for all of the above factors in *Arabidopsis* and rice. We excluded CLM paralogs in this analysis, because, mechanistically, the CLM paralogs are both lowly methylated, thus their genetic and expression divergences are less likely controlled by methylation. In contrast, expression of CHM paralogs would have a stronger effect imposed by DNA methylation. Indeed, we observed that CLM paralogs have different expression divergence pattern (significant higher expression divergence) from CHM paralogs. If we pooled CLM and CHM paralogs together to compute the correlation between methylation conservation and expression divergence, the correlation would contain noises and could not stand out. We found that the partial correlations are significantly negative in both genomes (Spearman’s rank correlation *p*<0.05, Table 4 and also see Figure S2). Further, the two proxies of expression divergence, e.g. expression level and specificity divergence, positively correlated with each other (Spearman’s rank correlation coefficient 0.3551, *p*<2.2e-16), and methylation conservation has a stronger negative correlation with expression level change than specificity difference (Table 4). Overall, these results indicated that methylation divergence does associate with expression divergence of duplicate genes.

**Table 4:** Spearman correlations between methylation conservation and expression divergence with other factors simultaneously controlled.

Species	Expression level divergence			
*Arabidopsis*	Partial coef	*p* value	General coef	*p* value
Methylation level	−0.1405	2.003e-06	−0.1431	4.1e-05
Methylation pattern	−0.0955	0.0038	−0.0815	0.0355
***Arabidopsis***	**Expression specificity divergence**			
	**Partial coef**	***p*** ** value**	**General coef**	***p*** ** value**
Methylation level	−0.0928	0.0081	−0.1135	0.0012
Methylation pattern	−0.0922	0.0177	−0.1007	0.0093
	**Expression level divergence**			
**Rice**	**Partial coef**	***p*** ** value**	**General coef**	***p*** ** value**
Methylation level	−0.1141	0.0014	−0.1524	2.048e-05
Methylation pattern	−0.1333	9.135e-04	−0.1410	4.451e-04

Note: Correlation coefficient and p value was computed with spearman correlation.

### Methylation level divergence is overall more important for expression divergence of paralogs than methylation pattern divergence

**Figure 8:**
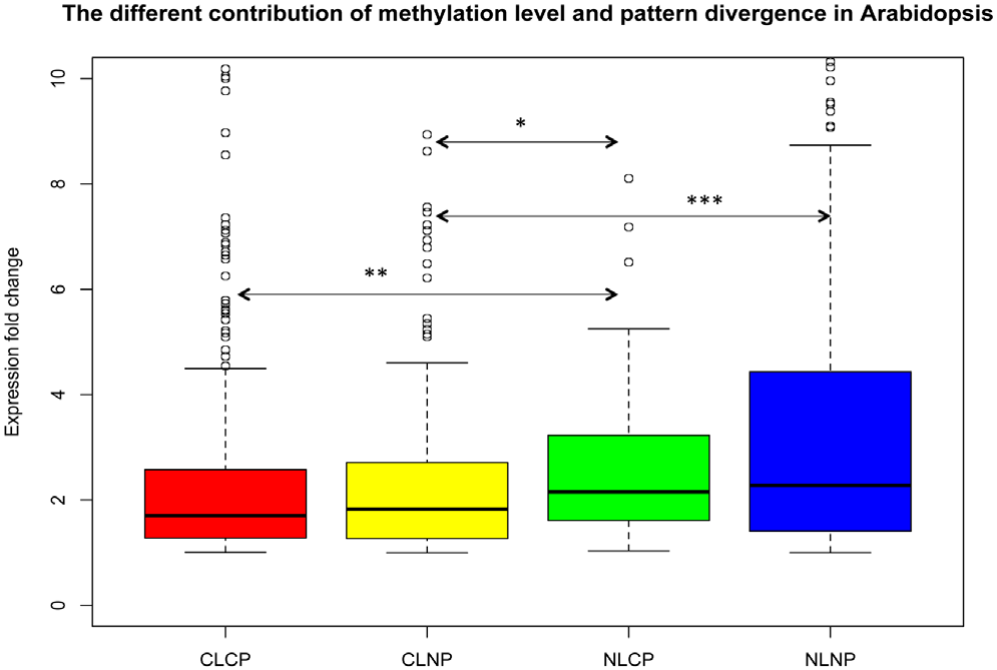
Differential contributions of methylation level and pattern divergence to expression level divergence of *Arabidopsis* paralogs. CLCP: paralogs with Conserved high methylation Level and Pattern. CLNP: paralogs with Conserved high methylation Level and Non-conserved methylation Pattern. NLCP: paralogs with Non-conserved methylation Level and Conserved high methylation Pattern. NLNP: paralogs with Non-conserved methylation Level and Pattern. Significant levels of Wilcoxon rank sum test were shown as “*”: *p*<0.05, “**”: *p*<0.01, and “***”: *p*<0.001.

To determine whether divergence of methylation level or pattern is more relevant to expression divergence of paralogs, we classified the paralogs into the following four categories: 1) Conserved high methylation Level and Pattern (CLCP); 2) Conserved high methylation Level and Non-conserved methylation Pattern (CLNP); 3) Non-conserved methylation Level and Conserved high methylation Pattern (NLCP); and 4) Non-conserved methylation Level and Pattern (NLNP). We found that in *Arabidopsis* there was no significant difference between the expression level changes of CLCP and CLNP paralogs, as well as between those of NLCP and NLNP ones (Figure 8). However, the expression changes of NLCP and NLNP paralogs are significant larger than those of CLCP and CLNP ones, respectively. Furthermore, the expression changes of NLCP ones are significantly larger than those of CLNP ones (Figure 8). This observation suggests that methylation level divergence overall is more relevant to expression change of paralogs than methylation pattern divergence in *Arabidopsis*.

### Exonic regions contribute more to gene body methylation divergence than intronic regions

To estimate whether the methylation level divergence in exonic and intronic regions contribute equally to the gene body methylation divergence, we divided the paralogs into the following categories using previous approaches: 1) conserved methylation level (CM) in exonic regions; 2) non-conserved methylation level (NCM) in exonic regions; 3) CM in intronic regions; 4) NCM in intronic regions. With Fisher test, we found that the proportion of exonic NCM among exonic NCM+CM was significantly higher than that of intronic regions in *Arabidopsis* and rice (Table 5, Fisher test, *Arabidopsis*: *p*<2.2e-16; rice: p  = 2.212e-10), suggesting exonic methylation divergence may contribute more to gene body methylation divergence than intronic methylation. Further, we found exonic methylation divergence is more closely correlated with expression divergence than intronic methylation divergence (Table S11), suggesting the exonic methylation divergence may play a more important role in the association with gene expression compared to intronic methylation divergence, for example, through regulating the alternative-splicing of transcripts.

**Table 5:** Paralogs with CM and NCM in exonic and intronic regions.

	Exonic		Intronic		Exonic Intronic		
Species	CM	NCM	CM	NCM	NCM/(CM+NCM)		*p* value^a^
*Arabidopsis*	1741	601	1165	13	25.66%	1.10%	<2.2e-16
Rice	1435	693	1214	367	32.57%	23.21%	2.212e-10

a *p* value was computed with Fisher test.

### Dispersed duplicate genes tend to have more divergent DNA methylation level/pattern

At the end, we explored the potential mechanisms contributing to the differential methylation between paralogs. Previous studies showed that DNA-based dispersed duplications in *Arabidopsis* tend to differ in DNA methylation in promoter regions than other gene duplication mechanisms [89]. In rice, different duplication mechanisms generate paralogs with different physical distance; which are associated with different body methylation level conservation [51]. Our analyses indicated that DNA-based dispersed gene duplication (Fisher test: methylation level: *p* = 2.69e-05; pattern: *p* = 0.0074) and RNA-based retrotransposition (Fisher test: methylation level: *p* = 0.06467) generated a higher proportion of paralogs with divergent methylation level and/or pattern than tandem and segmental duplication in rice. We also found the methylation level (Wilcoxon rank sum test, *p* = 3.248e-06) and pattern (Wilcoxon rank sum test, *p* = 1.689e-9) of segmental duplication is more conserved than those of the duplicate genes generated by single events, such as tandem duplication, dispersed duplication in rice. However, we did not observe such pattern in *Arabidopsis*, which might be due to the intrinsic methylation property of *Arabidopsis* genome. And the underlying mechanism remains further to be explored.

Previous studies have demonstrated that various mechanisms contributed to the methylation variation in trans-generational epialleles, and indicated that DNA methylation could spread from highly methylated transposable elements (TEs) and repetitive sequences to the nearby genes [3], [4], [77], [85], [90], [91]. Given our discovery that dispersed duplications generate a higher proportion of divergently methylated paralogs in rice, we asked whether the dispersed paralogs are more frequently associated with asymmetric TE/repeat sequence distribution, namely one duplicate copy flanked with TE/repeat sequence but not the other in rice. This could lead to the one flanked by TE/repeat sequence being more likely to be methylated, while the other without flanking TE/repeat sequence was not. Interestingly, we found that DNA-level dispersed duplication and retrotransposition paralogs were more frequently associated with asymmetric TE/repeat sequence distribution than tandem and segmental duplication ones in rice (Fisher test, *p*<0.05). The same trend was observed when we used different cutoff length of paralog flanking regions to search for the nearby TEs (Table S12).

## Discussion and Conclusions

In human, a exclusive relationship of gene body DNA methylation with evolutionary and expression divergence of paralogs could not be revealed [48]. Our analysis in plants added a different aspect regarding with the property and significant role of gene body DNA methylation in gene duplication. Gene body methylation is a pervasive phenomenon in various species and their pattern is highly conserved in orthologs from highly diverged species [23], [28], [34], [79], [92], [93]. Furthermore, as demonstrated previously, gene body DNA methylation in plant genomes has been considered as the main mode of DNA methylation and links with gene transcription [34]. Expectedly, the divergence of gene body methylation could play a functional role in influencing the evolution and divergence of paralogs given the interplay between gene transcription and DNA methylation [26], [29], [30], [94]. Specifically, paralogs initially tended to maintain same expression pattern and retained similar function; and gene body DNA methylation could regulate gene expression. Therefore, the divergence of gene body DNA methylation could provide a fast trajectory to differentiate gene expression of paralogs, leading to the divergence and thus the preservation of duplicate genes [52].

To validate the association between gene body DNA methylation and divergence of plant duplicate genes and the contribution of methylation to the divergence of plant duplicate genes, we analyzed a large number of reliable paralogs spanning different evolutionary ages from two distantly related plant species, *Arabidopsis* and *Oryza*, which represent dicots and monocots, respectively. We first showed that divergence of methylation level was positively correlated to the genetic evolution of paralogs in terms of Ks and Ka. This result suggested that methylation level variation accumulates along evolutionary time, and companies with neutral DNA mutation and amino acid changes. These postulations are supported by the previous discoveries that CG-SMP (single methylation polymorphism)-based phylogenetic tree is correlated to the SNP-based phylogenetic tree among multiple populations of *Arabidopsis* or closely related rice species [27], [77].

Differential gene body DNA methylation covaries with gene expression divergence between duplicate genes. It has been hypothesized that epigenetic modification of paralogs could facilitate functional divergence of duplicate genes [52]. Consistently, we demonstrated that conserved highly methylated duplicate genes have lower expression level and specificity divergence than conserved lowly- or non-conserved methylated duplicate genes. Remarkably, this pattern still robustly exists even after we controlled other factors known to affect gene expression divergence. Our observation is reflecting with previous trans-generational epigenetic variation studies, which indicated that epigenetic variation could contribute to phenotypic diversity [85], [90] and that *de novo* genes could originate through change the DNA methylation status (Silveira, et al. 2013). However, two additional considerations should be explored futher. First, although we quantitatively controlled for other factors, we could not controll for all the factors completely. For example, the same amount of nucleotide subsititions occuring in the different gene regions might also contribute to expression divergence of paralogs, and the data available for *cis*- and post-transcription regulation are limited. Also, gene body methylation might interplay with other mechanisms together and contribute to the divergence of duplicate genes.

Methylation level measures the overall methyaltion density of genes. Whereas methylation pattern measures methylation property of specific cytocine sites of genes. Therefore, methylation level and pattern divergence could behave differentially in affecting the expression divergence. We identified the substantial roles of DNA methylation level for expression and genetic divergence of *Arabidopsis* duplicate genes. This result supports the hypothesis that gene body methylation might be maintained as a property of regions with a minimal/maximal methylation level over the whole region while experiencing site to site methylation stochasticity, driven by natural selection [34]. Alternatively, methylation changes in specific sites could also affect gene expression. For example, it was previously found that enhancer access could be modulated by a single methylation event [24].

The methylation divergence of duplicate genes may be the outcome of spontaneous methylation variation, which might be impacted by natural selection (see reviews by [95]–[97]). A few factors could contribute to the methylation divergence of paralogs. For example, methylation divergence from exons and introns could differently contribute to the divergence of gene body methylation in duplicate genes. We found that more methylation divergence occurs in the exonic regions than intronic regions. Previously, two studies reported conflicting results about the involvement of exons and introns in differentially methylated regions (DMR) among different *Arabidopsis* lines due to spontaneous epigenetic variation. Becker et al. (2011) showed that CG-DMRs in exons were more abundant than those in introns [85], while Schmitz et al. (2011) found the opposite pattern [90]. Previous studies also showed that methylation in exons may be associated with mRNA-splicing, thus exonic methylation divergence might be relevant to the mRNA-splicing divergence and expression divergence of paralogs [29], [98].

We demonstrated that exonic methylation divergence is more closely correlated with expression divergence than intronic methylation divergence, which implies that the exonic methylation divergence might have more functional consequences than intronic methylation divergence. Conclusively, exonic methylation variation might have a higher chance to be maintained than intronic methylation variation, which can explain our observation that exonic regions bear more methylation divergence than intronic regions. However, it remains to be explored other functional roles of methylation in exonic and intronic regions. Furthermore, the genomic environments of duplicate genes can also impact the divergence of methylation among paralogs. Our analysis confirmed that the physical distance of paralogs negatively correlated with the conservation of methylation level and pattern, and that dispersed duplicate genes more tend to have divergent methylation level and pattern.

We found inconsistent patterns, for some analyses, in *Arabidopsis* and rice. This may be due to two factors. First, the limitation of availability and the quality of the data in two genomes are different. For example, the expression data of *Arabidopsis* were obtained from RNA-seq but those of rice were generated from DGE. Second, properties of methylation in the two genomes are different. For example, the methylation level in rice is at least two-fold higher than that in *Arabidopsis*.

In conclusion, the correlation between the divergence of gene body DNA methylation and expression in plant duplicate genes implicates that gene body DNA methylation could serve as another avenue for duplicate genes to develop different expression and undergo different evolutionary fates. Our study indicates that gene body DNA methylation as one type of epigenetic modifications is an important facilitator that potentially drove divergence and evolution of plant duplicate genes.

## Supporting Information

Figure S1 **Plots of methylation level** ***vs*** **. Ka and Ks for duplicate genes in** ***Arabidopsis*** **and rice.** Red line is the linear regression fit to the data, which were generated with *lm* function in R.
(TIF)

Figure S2 **Plots of the methylation level** ***vs.*** **the expression level for duplicate genes in** ***Arabidopsis*** **and rice.** Red line is the linear regression fit to the data, which were generated with *lm* function in R.
(TIF)

Table S1 **Reprocessed error rate in un-methylated chloroplast genome and methylation data for** ***Arabidopsis*** **and rice.**
(PDF)

Table S2 **The correlation between methylation level and gene length/exon number.**
(PDF)

Table S3 **The correlation between methylation level and expression level/specificity in** ***Arabidopsis.***
(PDF)

Table S4 **The correlation between expression level and other factors.**
(PDF)

Table S5 **The correlation between methylation level and theta in** ***Arabidopsis.***
(PDF)

Table S6 **The correlation between theta and other factors.**
(PDF)

Table S7 **The relationship of methylation conservation and expression divergence that is corresponded to** **Figure 6** **.**
(PDF)

Table S8 **The relationship of methylation conservation and expression divergence with gene structure controlled.**
(PDF)

Table S9 **The relationship of methylation conservation and expression divergence with nucleotide substitution rate controlled.**
(PDF)

Table S10 **The relationship of methylation conservation and expression divergence with small RNA abundance controlled.**
(PDF)

Table S11 **The correlation between exonic/intronic methylation divergence and expression level divergence.**
(PDF)

Table S12 **The p-values corresponding to the different flanking region length cutoffs in searching nearby TEs.**
(PDF)

## References

[1] Cubas P, Vincent C, Coen E (1999) An epigenetic mutation responsible for natural variation in floral symmetry. Nature 401: 157–161. 10490023 10.1038/43657

[2] Manning K, Tor M, Poole M, Hong Y, Thompson AJ, et al (2006) A naturally occurring epigenetic mutation in a gene encoding an SBP-box transcription factor inhibits tomato fruit ripening. Nat Genet 38: 948–952. 16832354 10.1038/ng1841

[3] Martin A, Troadec C, Boualem A, Rajab M, Fernandez R, et al (2009) A transposon-induced epigenetic change leads to sex determination in melon. Nature 461: 1135–1138. 19847267 10.1038/nature08498

[4] Bender J, Fink GR (1995) Epigenetic control of an endogenous gene family is revealed by a novel blue fluorescent mutant of Arabidopsis. Cell 83: 725–734. 8521489 10.1016/0092-8674(95)90185-x

[5] Law JA, Jacobsen SE (2010) Establishing, maintaining and modifying DNA methylation patterns in plants and animals. Nat Rev Genet 11: 204–220. 20142834 10.1038/nrg2719 PMC3034103

[6] Henderson IR, Jacobsen SE (2007) Epigenetic inheritance in plants. Nature 447: 418–424. 17522675 10.1038/nature05917

[7] Wassenegger M, Heimes S, Riedel L, Sanger HL (1994) Rna-Directed De-Novo Methylation of Genomic Sequences in Plants. Cell 76: 567–576. 8313476 10.1016/0092-8674(94)90119-8

[8] Matzke M, Kanno T, Claxinger L, Huettel B, Matzke AJM (2009) RNA-mediated chromatin-based silencing in plants. Current Opinion in Cell Biology 21: 367–376. 19243928 10.1016/j.ceb.2009.01.025

[9] Vongs A, Kakutani T, Martienssen RA, Richards EJ (1993) Arabidopsis-Thaliana DNA Methylation Mutants. Science 260: 1926–1928. 8316832 10.1126/science.8316832

[10] Bartee L, Malagnac F, Bender J (2001) Arabidopsis cmt3 chromomethylase mutations block non-CG methylation and silencing of an endogenous gene. Genes & Development 15: 1753–1758. 11459824 10.1101/gad.905701 PMC312734

[11] Lindroth AM, Cao XF, Jackson JP, Zilberman D, McCallum CM, et al (2001) Requirement of CHROMOMETHYLASE3 for maintenance of CpXpG methylation. Science 292: 2077–2080. 11349138 10.1126/science.1059745

[12] Jullien PE, Mosquna A, Ingouff M, Sakata T, Ohad N, et al (2008) Retinoblastoma and its binding partner MSI1 control imprinting in Arabidopsis. PLoS Biol 6: e194. 18700816 10.1371/journal.pbio.0060194 PMC2504488

[13] Choi YH, Gehring M, Johnson L, Hannon M, Harada JJ, et al (2002) DEMETER, a DNA glycosylase domain protein, is required for endosperm gene imprinting and seed viability in Arabidopsis. Cell 110: 33–42. 12150995 10.1016/s0092-8674(02)00807-3

[14] Gong ZH, Morales-Ruiz T, Ariza RR, Roldan-Arjona T, David L, et al (2002) ROS1, a repressor of transcriptional gene silencing in Arabidopsis, encodes a DNA glycosylase/lyase. Cell 111: 803–814. 12526807 10.1016/s0092-8674(02)01133-9

[15] Bird A (2002) DNA methylation patterns and epigenetic memory. Genes Dev 16: 6–21. 11782440 10.1101/gad.947102

[16] Hsieh TF, Shin J, Uzawa R, Silva P, Cohen S, et al (2011) Regulation of imprinted gene expression in Arabidopsis endosperm. Proc Natl Acad Sci U S A 108: 1755–1762. 21257907 10.1073/pnas.1019273108 PMC3033266

[17] Dowen RH, Pelizzola M, Schmitz RJ, Lister R, Dowen JM, et al (2012) Widespread dynamic DNA methylation in response to biotic stress. Proc Natl Acad Sci U S A 109: E2183–2191. 22733782 10.1073/pnas.1209329109 PMC3420206

[18] Lisch D (2009) Epigenetic regulation of transposable elements in plants. Annu Rev Plant Biol 60: 43–66. 19007329 10.1146/annurev.arplant.59.032607.092744

[19] Morgan HD, Santos F, Green K, Dean W, Reik W (2005) Epigenetic reprogramming in mammals. Hum Mol Genet 14 Spec No 1: R47–58. 10.1093/hmg/ddi114 15809273

[20] Feng J, Fouse S, Fan G (2007) Epigenetic regulation of neural gene expression and neuronal function. Pediatr Res 61: 58R–63R. 10.1203/pdr.0b013e3180457635 17413844

[21] Kulis M, Heath S, Bibikova M, Queiros AC, Navarro A, et al (2012) Epigenomic analysis detects widespread gene-body DNA hypomethylation in chronic lymphocytic leukemia. Nat Genet 44: 1236–1242. 23064414 10.1038/ng.2443

[22] Diez CM, Roessler K, Gaut BS (2014) Epigenetics and plant genome evolution. Curr Opin Plant Biol 18: 1–8. 24424204 10.1016/j.pbi.2013.11.017

[23] Suzuki MM, Bird A (2008) DNA methylation landscapes: provocative insights from epigenomics. Nat Rev Genet 9: 465–476. 18463664 10.1038/nrg2341

[24] Bell AC, Felsenfeld G (2000) Methylation of a CTCF-dependent boundary controls imprinted expression of the Igf2 gene. Nature 405: 482–485. 10839546 10.1038/35013100

[25] Zhang X, Yazaki J, Sundaresan A, Cokus S, Chan SW, et al (2006) Genome-wide high-resolution mapping and functional analysis of DNA methylation in arabidopsis. Cell 126: 1189–1201. 16949657 10.1016/j.cell.2006.08.003

[26] Zilberman D, Gehring M, Tran RK, Ballinger T, Henikoff S (2007) Genome-wide analysis of Arabidopsis thaliana DNA methylation uncovers an interdependence between methylation and transcription. Nat Genet 39: 61–69. 17128275 10.1038/ng1929

[27] Li X, Zhu J, Hu F, Ge S, Ye M, et al (2012) Single-base resolution maps of cultivated and wild rice methylomes and regulatory roles of DNA methylation in plant gene expression. BMC Genomics 13: 300. 22747568 10.1186/1471-2164-13-300 PMC3447678

[28] Zemach A, McDaniel IE, Silva P, Zilberman D (2010) Genome-wide evolutionary analysis of eukaryotic DNA methylation. Science 328: 916–919. 20395474 10.1126/science.1186366

[29] Shukla S, Kavak E, Gregory M, Imashimizu M, Shutinoski B, et al (2011) CTCF-promoted RNA polymerase II pausing links DNA methylation to splicing. Nature 479: 74–79. 21964334 10.1038/nature10442 PMC7398428

[30] Maunakea AK, Nagarajan RP, Bilenky M, Ballinger TJ, D'Souza C, et al (2010) Conserved role of intragenic DNA methylation in regulating alternative promoters. Nature 466: 253–257. 20613842 10.1038/nature09165 PMC3998662

[31] Teixeira FK, Colot V (2009) Gene body DNA methylation in plants: a means to an end or an end to a means? Embo Journal 28: 997–998. 19384348 10.1038/emboj.2009.87 PMC2683714

[32] Roudier F, Teixeira FK, Colot V (2009) Chromatin indexing in Arabidopsis: an epigenomic tale of tails and more. Trends in Genetics 25: 511–517. 19850370 10.1016/j.tig.2009.09.013

[33] Takuno S, Gaut BS (2012) Body-Methylated Genes in Arabidopsis thaliana Are Functionally Important and Evolve Slowly. Mol Biol Evol 29: 219–227. 21813466 10.1093/molbev/msr188

[34] Takuno S, Gaut BS (2013) Gene body methylation is conserved between plant orthologs and is of evolutionary consequence. Proc Natl Acad Sci U S A 110: 1797–1802. 23319627 10.1073/pnas.1215380110 PMC3562806

[35] Ohno S (1970) Evolution by gene duplication. Berlin, New York,: Springer-Verlag. xv, 160 p.

[36] Ohta T (1973) Slightly deleterious mutant substitutions in evolution. Nature 246: 96–98. 4585855 10.1038/246096a0

[37] Harada E, Nakagawa J, Asano T, Taoka M, Sorimachi H, et al (2012) Functional evolution of duplicated odorant-binding protein genes, Obp57d and Obp57e, in Drosophila. PLoS One 7: e29710. 22238638 10.1371/journal.pone.0029710 PMC3253112

[38] Piatigorsky J, Wistow G (1991) The recruitment of crystallins: new functions precede gene duplication. Science 252: 1078–1079. 2031181 10.1126/science.252.5009.1078

[39] Ferris SD, Whitt GS (1979) Evolution of the differential regulation of duplicate genes after polyploidization. J Mol Evol 12: 267–317. 448746 10.1007/BF01732026

[40] Hittinger CT, Carroll SB (2007) Gene duplication and the adaptive evolution of a classic genetic switch. Nature 449: 677–681. 17928853 10.1038/nature06151

[41] Force A, Lynch M, Pickett FB, Amores A, Yan YL, et al (1999) Preservation of duplicate genes by complementary, degenerative mutations. Genetics 151: 1531–1545. 10101175 10.1093/genetics/151.4.1531 PMC1460548

[42] Lynch M, Conery JS (2003) The origins of genome complexity. Science 302: 1401–1404. 14631042 10.1126/science.1089370

[43] Lynch M, O'Hely M, Walsh B, Force A (2001) The probability of preservation of a newly arisen gene duplicate. Genetics 159: 1789–1804. 11779815 10.1093/genetics/159.4.1789 PMC1461922

[44] Shiu SH, Byrnes JK, Pan R, Zhang P, Li WH (2006) Role of positive selection in the retention of duplicate genes in mammalian genomes. Proc Natl Acad Sci U S A 103: 2232–2236. 16461903 10.1073/pnas.0510388103 PMC1413713

[45] Hughes AL (1994) The evolution of functionally novel proteins after gene duplication. Proc Biol Sci 256: 119–124. 8029240 10.1098/rspb.1994.0058

[46] Chang AY, Liao BY (2012) DNA methylation rebalances gene dosage after mammalian gene duplications. Mol Biol Evol 29: 133–144. 21821837 10.1093/molbev/msr174

[47] Li J, Musso G, Zhang Z (2008) Preferential regulation of duplicated genes by microRNAs in mammals. Genome Biol 9: R132. 18727826 10.1186/gb-2008-9-8-r132 PMC2575522

[48] Keller TE, Yi SV (2014) DNA methylation and evolution of duplicate genes. Proc Natl Acad Sci U S A 111: 5932–5937. 24711408 10.1073/pnas.1321420111 PMC4000835

[49] Widman N, Jacobsen SE, Pellegrini M (2009) Determining the conservation of DNA methylation in Arabidopsis. Epigenetics 4: 119–124. 19384058 10.4161/epi.4.2.8214

[50] Wang J, Marowsky NC, Fan C (2013) Divergent evolutionary and expression patterns between lineage specific new duplicate genes and their parental paralogs in Arabidopsis thaliana. PLoS One 8: e72362. 24009676 10.1371/journal.pone.0072362 PMC3756979

[51] Wang Y, Wang X, Lee TH, Mansoor S, Paterson AH (2013) Gene body methylation shows distinct patterns associated with different gene origins and duplication modes and has a heterogeneous relationship with gene expression in Oryza sativa (rice). New Phytol 198: 274–283. 23356482 10.1111/nph.12137

[52] Rodin SN, Riggs AD (2003) Epigenetic silencing may aid evolution by gene duplication. J Mol Evol 56: 718–729. 12911035 10.1007/s00239-002-2446-6

[53] Rapp RA, Wendel JF (2005) Epigenetics and plant evolution. New Phytol 168: 81–91. 16159323 10.1111/j.1469-8137.2005.01491.x

[54] Chen ZJ, Ni Z (2006) Mechanisms of genomic rearrangements and gene expression changes in plant polyploids. Bioessays 28: 240–252. 16479580 10.1002/bies.20374 PMC1986666

[55] Lister R, O'Malley RC, Tonti-Filippini J, Gregory BD, Berry CC, et al (2008) Highly integrated single-base resolution maps of the epigenome in Arabidopsis. Cell 133: 523–536. 18423832 10.1016/j.cell.2008.03.029 PMC2723732

[56] Zemach A, Kim MY, Silva P, Rodrigues JA, Dotson B, et al (2010) Local DNA hypomethylation activates genes in rice endosperm. Proc Natl Acad Sci U S A 107: 18729–18734. 20937895 10.1073/pnas.1009695107 PMC2972920

[57] Schmid M, Davison TS, Henz SR, Pape UJ, Demar M, et al (2005) A gene expression map of Arabidopsis thaliana development. Nature Genetics 37: 501–506. 15806101 10.1038/ng1543

[58] Jeong DH, Park S, Zhai JX, Gurazada SGR, De Paoli E, et al (2011) Massive Analysis of Rice Small RNAs: Mechanistic Implications of Regulated MicroRNAs and Variants for Differential Target RNA Cleavage. Plant Cell 23: 4185–4207. 22158467 10.1105/tpc.111.089045 PMC3269859

[59] Krueger F, Andrews SR (2011) Bismark: a flexible aligner and methylation caller for Bisulfite-Seq applications. Bioinformatics 27: 1571–1572. 21493656 10.1093/bioinformatics/btr167 PMC3102221

[60] Fojtova M, Kovarik A, Matyasek R (2001) Cytosine methylation of plastid genome in higher plants. Fact or artefact? Plant Science 160: 585–593. 11448733 10.1016/s0168-9452(00)00411-8

[61] Storey JD (2003) The positive false discovery rate: A Bayesian interpretation and the q-value. Annals of Statistics 31: 2013–2035.

[62] Storey JD, Tibshirani R (2003) Statistical significance for genomewide studies. Proceedings of the National Academy of Sciences of the United States of America 100: 9440–9445. 12883005 10.1073/pnas.1530509100 PMC170937

[63] Storey JD (2002) A direct approach to false discovery rates. Journal of the Royal Statistical Society Series B-Statistical Methodology 64: 479–498.

[64] Langmead B, Trapnell C, Pop M, Salzberg SL (2009) Ultrafast and memory-efficient alignment of short DNA sequences to the human genome. Genome Biol 10: R25. 19261174 10.1186/gb-2009-10-3-r25 PMC2690996

[65] Trapnell C, Williams BA, Pertea G, Mortazavi A, Kwan G, et al (2010) Transcript assembly and quantification by RNA-Seq reveals unannotated transcripts and isoform switching during cell differentiation. Nat Biotechnol 28: 511–U174. 20436464 10.1038/nbt.1621 PMC3146043

[66] Yanai I, Benjamin H, Shmoish M, Chalifa-Caspi V, Shklar M, et al (2005) Genome-wide midrange transcription profiles reveal expression level relationships in human tissue specification. Bioinformatics 21: 650–659. 15388519 10.1093/bioinformatics/bti042

[67] Li H, Durbin R (2009) Fast and accurate short read alignment with Burrows-Wheeler transform. Bioinformatics 25: 1754–1760. 19451168 10.1093/bioinformatics/btp324 PMC2705234

[68] Kent WJ (2002) BLAT–the BLAST-like alignment tool. Genome Res 12: 656–664. 11932250 10.1101/gr.229202 PMC187518

[69] Yang Z (2007) PAML 4: phylogenetic analysis by maximum likelihood. Mol Biol Evol 24: 1586–1591. 17483113 10.1093/molbev/msm088

[70] Katoh K, Kuma K, Toh H, Miyata T (2005) MAFFT version 5: improvement in accuracy of multiple sequence alignment. Nucleic Acids Res 33: 511–518. 15661851 10.1093/nar/gki198 PMC548345

[71] Cao J, Schneeberger K, Ossowski S, Gunther T, Bender S, et al (2011) Whole-genome sequencing of multiple Arabidopsis thaliana populations. Nat Genet 43: 956–963. 21874002 10.1038/ng.911

[72] Nei M, Li WH (1979) Mathematical model for studying genetic variation in terms of restriction endonucleases. Proc Natl Acad Sci U S A 76: 5269–5273. 291943 10.1073/pnas.76.10.5269 PMC413122

[73] Smit AFA, Hubley R, Green P (1996–2010) RepeatMasker Open-3.0.

[74] Kim SH, Yi SV (2007) Understanding relationship between sequence and functional evolution in yeast proteins. Genetica 131: 151–156. 17160620 10.1007/s10709-006-9125-2

[75] Kim SH, Yi SV (2006) Correlated asymmetry of sequence and functional divergence between duplicate proteins of Saccharomyces cerevisiae. Mol Biol Evol 23: 1068–1075. 16510556 10.1093/molbev/msj115

[76] Whittaker J (1996) Graphical models in applied multivariate statistics. New York: John Wiley and Sons.

[77] Schmitz RJ, Schultz MD, Urich MA, Nery JR, Pelizzola M, et al (2013) Patterns of population epigenomic diversity. Nature 495: 193–198. 23467092 10.1038/nature11968 PMC3798000

[78] Hua Z, Pool JE, Schmitz RJ, Schultz MD, Shiu SH, et al (2013) Epigenomic programming contributes to the genomic drift evolution of the F-Box protein superfamily in Arabidopsis. Proc Natl Acad Sci U S A 110: 16927–16932. 24082131 10.1073/pnas.1316009110 PMC3801079

[79] Sarda S, Zeng J, Hunt BG, Yi SV (2012) The evolution of invertebrate gene body methylation. Mol Biol Evol 29: 1907–1916. 22328716 10.1093/molbev/mss062

[80] Flores K, Wolschin F, Corneveaux JJ, Allen AN, Huentelman MJ, et al (2012) Genome-wide association between DNA methylation and alternative splicing in an invertebrate. BMC Genomics 13: 480. 22978521 10.1186/1471-2164-13-480 PMC3526459

[81] Watterson GA (1975) On the number of segregating sites in genetical models without recombination. Theor Popul Biol 7: 256–276. 1145509 10.1016/0040-5809(75)90020-9

[82] Pfeifer GP (2006) Mutagenesis at methylated CpG sequences. DNA Methylation: Basic Mechanisms 301: 259–281. 10.1007/3-540-31390-7_10 16570852

[83] Messeguer R, Ganal MW, Steffens JC, Tanksley SD (1991) Characterization of the Level, Target Sites and Inheritance of Cytosine Methylation in Tomato Nuclear-DNA. Plant Molecular Biology 16: 753–770. 1859863 10.1007/BF00015069

[84] Bird AP (1980) DNA methylation and the frequency of CpG in animal DNA. Nucleic Acids Res 8: 1499–1504. 6253938 10.1093/nar/8.7.1499 PMC324012

[85] Becker C, Hagmann J, Muller J, Koenig D, Stegle O, et al (2011) Spontaneous epigenetic variation in the Arabidopsis thaliana methylome. Nature 480: 245–249. 22057020 10.1038/nature10555

[86] Arikit S, Zhai JX, Meyers BC (2013) Biogenesis and function of rice small RNAs from non-coding RNA precursors. Current Opinion in Plant Biology 16: 170–179. 23466255 10.1016/j.pbi.2013.01.006

[87] Johnson C, Kasprzewska A, Tennessen K, Fernandes J, Nan GL, et al (2009) Clusters and superclusters of phased small RNAs in the developing inflorescence of rice. Genome Research 19: 1429–1440. 19584097 10.1101/gr.089854.108 PMC2720183

[88] Wu L, Zhou HY, Zhang QQ, Zhang JG, Ni FR, et al (2010) DNA Methylation Mediated by a MicroRNA Pathway. Molecular Cell 38: 465–475. 20381393 10.1016/j.molcel.2010.03.008

[89] Wang Y, Wang X, Tang H, Tan X, Ficklin SP, et al (2011) Modes of gene duplication contribute differently to genetic novelty and redundancy, but show parallels across divergent angiosperms. PLoS One 6: e28150. 22164235 10.1371/journal.pone.0028150 PMC3229532

[90] Schmitz RJ, Schultz MD, Lewsey MG, O'Malley RC, Urich MA, et al (2011) Transgenerational Epigenetic Instability Is a Source of Novel Methylation Variants. Science 334: 369–373. 21921155 10.1126/science.1212959 PMC3210014

[91] Ahmed I, Sarazin A, Bowler C, Colot V, Quesneville H (2011) Genome-wide evidence for local DNA methylation spreading from small RNA-targeted sequences in Arabidopsis. Nucleic Acids Res 39: 6919–6931. 21586580 10.1093/nar/gkr324 PMC3167636

[92] Hunt BG, Brisson JA, Yi SV, Goodisman MA (2010) Functional conservation of DNA methylation in the pea aphid and the honeybee. Genome Biol Evol 2: 719–728. 20855427 10.1093/gbe/evq057 PMC2962555

[93] Zeng J, Yi SV (2010) DNA methylation and genome evolution in honeybee: gene length, expression, functional enrichment covary with the evolutionary signature of DNA methylation. Genome Biol Evol 2: 770–780. 20924039 10.1093/gbe/evq060 PMC2975444

[94] Jones PA (2012) Functions of DNA methylation: islands, start sites, gene bodies and beyond. Nat Rev Genet 13: 484–492. 22641018 10.1038/nrg3230

[95] Klironomos FD, Berg J, Collins S (2013) How epigenetic mutations can affect genetic evolution: model and mechanism. Bioessays 35: 571–578. 23580343 10.1002/bies.201200169

[96] Bonduriansky R (2012) Rethinking heredity, again. Trends Ecol Evol 27: 330–336. 22445060 10.1016/j.tree.2012.02.003

[97] Shea N, Pen I, Uller T (2011) Three epigenetic information channels and their different roles in evolution. J Evol Biol 24: 1178–1187. 21504495 10.1111/j.1420-9101.2011.02235.x PMC3116147

[98] Chodavarapu RK, Feng SH, Bernatavichute YV, Chen PY, Stroud H, et al (2010) Relationship between nucleosome positioning and DNA methylation. Nature 466: 388–392. 20512117 10.1038/nature09147 PMC2964354

