# Supplementary figures and images for: Divergence of Gene Body DNA Methylation and Evolution of Plant Duplicate Genes
Source: PLoS One. 2014 Oct 13;9(10):e110357. doi: 10.1371/journal.pone.0110357 (PMC4195714; doi:10.1371/journal.pone.0110357)

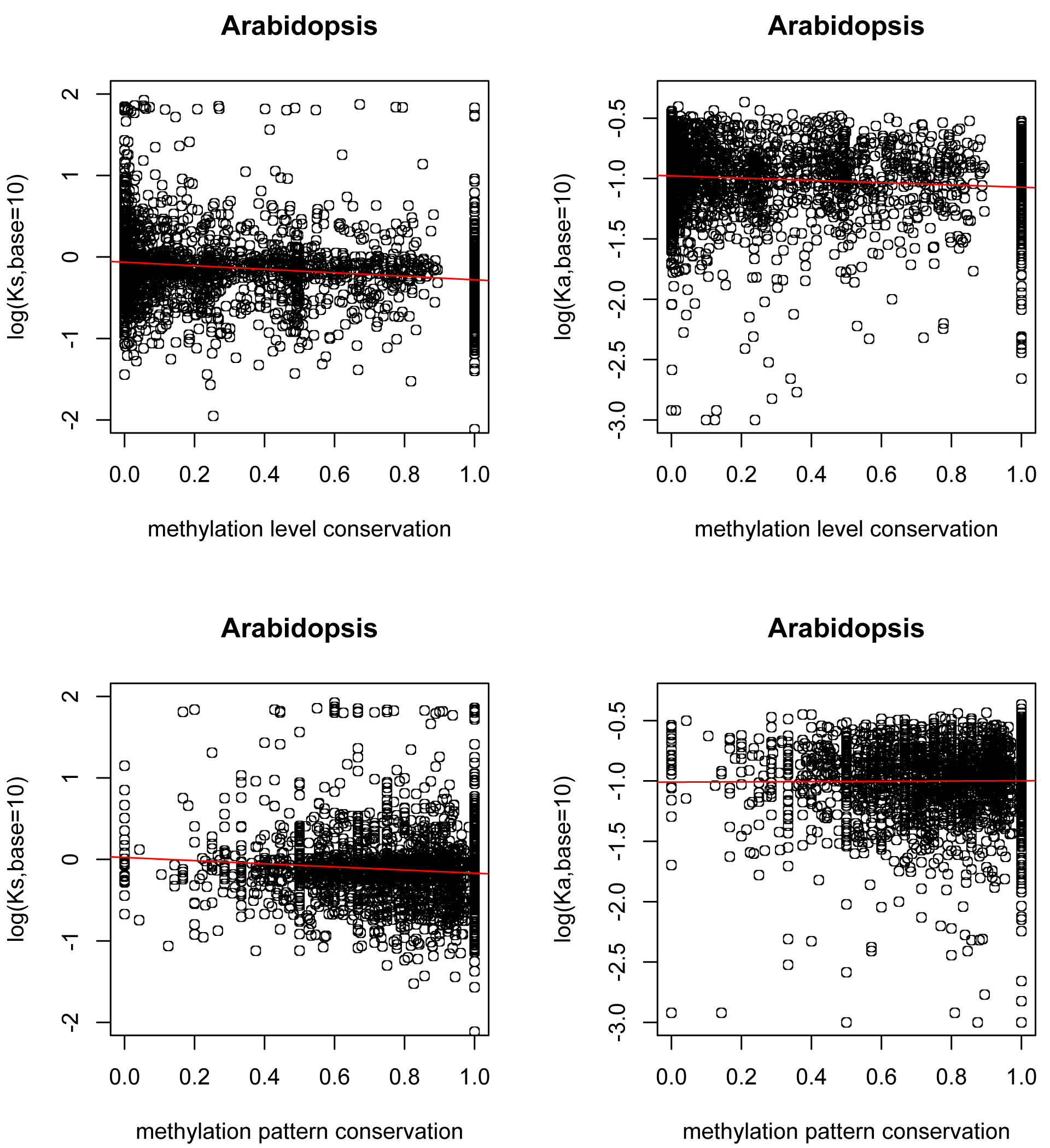

Supplement: Figure S1 — Plots of methylation level vs . Ka and Ks for duplicate genes in Arabidopsis and rice. Red line is the linear regression fit to the data, which were generated with lm function in R. (TIF) [file pone.0110357.s001.tif]

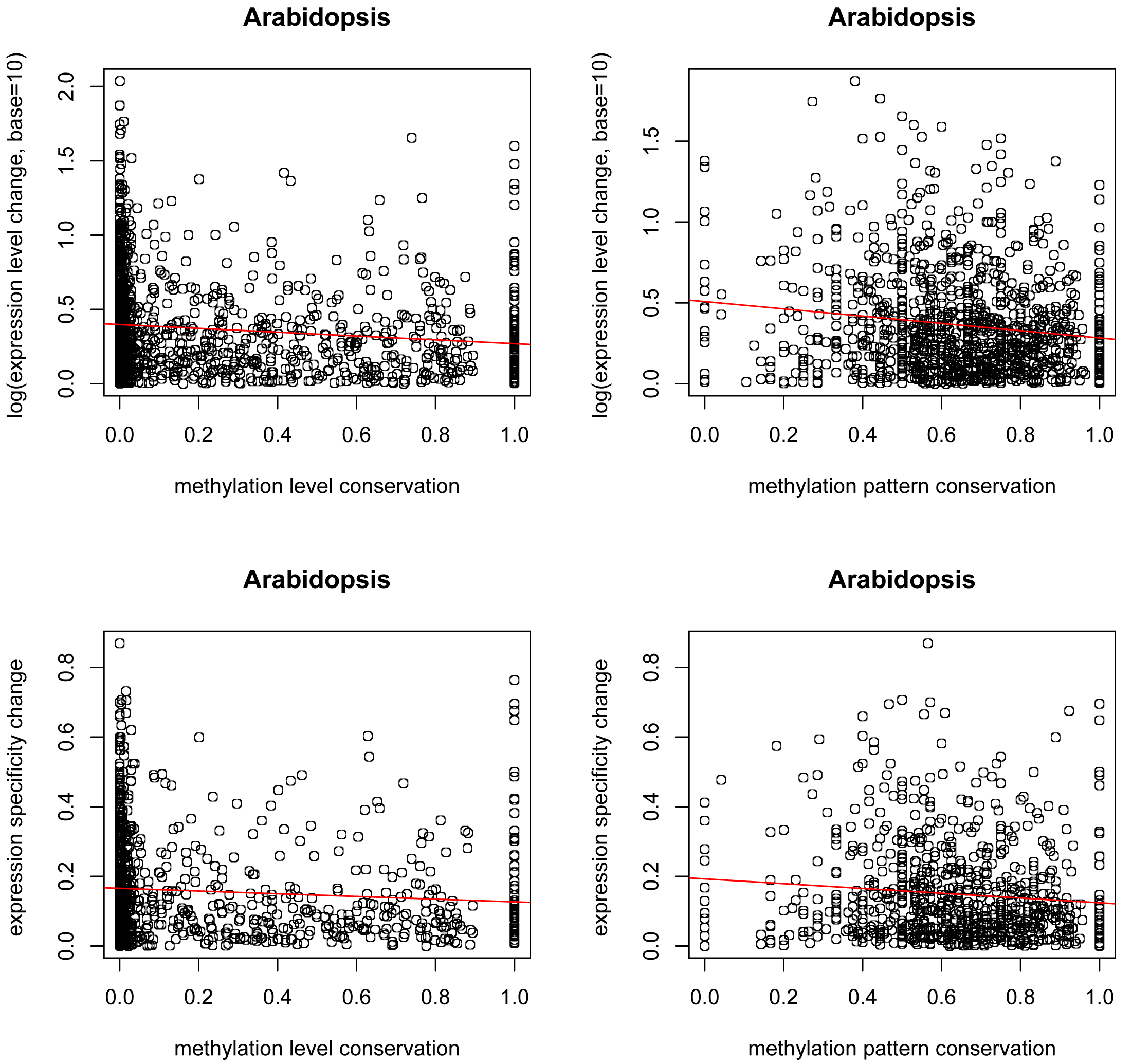

Supplement: Figure S2 — Plots of the methylation level vs. the expression level for duplicate genes in Arabidopsis and rice. Red line is the linear regression fit to the data, which were generated with lm function in R. (TIF) [file pone.0110357.s002.tif]
