# Supplementary material for: Divergence of Gene Body DNA Methylation and Evolution of Plant Duplicate Genes
Source: PLoS One. 2014 Oct 13;9(10):e110357. doi: 10.1371/journal.pone.0110357 (PMC4195714; doi:10.1371/journal.pone.0110357)
Supplement: Table S1 — Reprocessed error rate in un-methylated chloroplast genome and methylation data for Arabidopsis and rice. (PDF) [file pone.0110357.s003.pdf]

Table S1. Reprocessed error rate in unmethylated chloroplast genome and methylation data for *Arabidopsis* and rice

Sample	***Arabidopsis***		**Rice**	
Error rate	0.02204323		0.011205582	
Methylation	Methylcytosine number	Methylation level	Methylcytosine number	Methylation level
Total	2483801	6.50%	24023001	16.43%
CG	1432822	23.27%	12601670	38.21%
CHG	602325	10.79%	6154401	25.91%
CHH	448654	1.69%	5266930	5.89%
