# Supplementary material for: Divergence of Gene Body DNA Methylation and Evolution of Plant Duplicate Genes
Source: PLoS One. 2014 Oct 13;9(10):e110357. doi: 10.1371/journal.pone.0110357 (PMC4195714; doi:10.1371/journal.pone.0110357)
Supplement: Table S2 — The correlation between methylation level and gene length/exon number. (PDF) [file pone.0110357.s004.pdf]

Table S2. The correlation between methylation level and gene length/exon number

	Spearman coefficients	p value	linear regression coefficient	coefficient significance	R-squared	smoothing spline regression	R-squared
Methylation level < 0.6							
Arabidopsis						*Arabidopsis*	
Mxon number	0.4607	< 2.2e-16	14.61606	<2e-16	0.1975	exon number	0.2098
Gene length	0.5619	< 2.2e-16	0.7834	<2e-16	0.241	gene length	0.295
**Rice**						Rice	
Exon number	0.5411	< 2.2e-16	10.49574	<2e-16	0.201	exon number	0.2108
Gene length	0.6373	< 2.2e-16	0.952889	<2e-16	0.3073	gene length	0.3488
Methylation level ≥ 0.6							
Arabidopsis							
Exon number	-0.5743	< 2.2e-16	-23.892	<2e-16	0.3047		
Gene length	-0.7327	< 2.2e-16	-1.9963	<2e-16	0.3754		
**Rice**							
Exon number	-0.4526	< 2.2e-16	-13.921	<2e-16	0.1426		
Gene length	-0.5547	< 2.2e-16	-1.45459	<2e-16	0.2795		
