# Supplementary material for: Divergence of Gene Body DNA Methylation and Evolution of Plant Duplicate Genes
Source: PLoS One. 2014 Oct 13;9(10):e110357. doi: 10.1371/journal.pone.0110357 (PMC4195714; doi:10.1371/journal.pone.0110357)
Supplement: Table S3 — The correlation between methylation level and expression level/specificity in Arabidopsis. (PDF) [file pone.0110357.s005.pdf]

Table S3. The correlation between methylation level and expression level/specificity in *Arabidopsis*

	Spearman coefficients	*p* value	linear regression coefficient	coefficient significance	R-squared	smoothing spline regression	R-squared
Methylation level < 0.5							
Expression level	0.1608	< 2.2e-16	0.8	1.12E-11	0.007939	expression level	0.0123
Expression specificity	-0.2073	< 2.2e-16	-0.49343	<2e-16	0.02282	expression specificity	0.0276
Methylation level ≥ 0.5							
Expression level	-0.4077	< 2.2e-16	-1.74613	<2e-16	0.2873		
Expression specificity	0.1683	0.007181	0.75092	4.34E-06	0.08336		
