# Supplementary material for: Divergence of Gene Body DNA Methylation and Evolution of Plant Duplicate Genes
Source: PLoS One. 2014 Oct 13;9(10):e110357. doi: 10.1371/journal.pone.0110357 (PMC4195714; doi:10.1371/journal.pone.0110357)
Supplement: Table S4 — The correlation between expression level and other factors. (PDF) [file pone.0110357.s006.pdf]

Table S4. The correlation between expression level and other factors

Expression level			Expression specificity		
Factors	coeff	*p* value	factors	coeff	*p* value
24sRNA abundance ratio	-0.1867	< 2.2e-16	24sRNA_abundance_ratio	0.1521902	< 2.2e-16
21sRNA abundance ratio	-0.1212	< 2.2e-16	21sRNA_abundance_ratio	0.114965	< 2.2e-16
Gene length	0.3212684	< 2.2e-16	gene length	-0.1871944	< 2.2e-16
Exon number	0.2804281	< 2.2e-16	exon number	-0.2265048	< 2.2e-16
Promoter methylation	-0.0795	< 2.2e-16	promoter_methy	-0.03980729	7.77E-08
Gene body methylation >=0.5	-0.5223	< 2.2e-16	body methy >=0.5	0.2316631	< 2.2e-16
Gene body methylation < 0.5	0.245	< 2.2e-16	body methy < 0.5	-0.2292145	< 2.2e-16
Theta (θ)	-0.1987	< 2.2e-16	theta	0.1439884	< 2.2e-16
