# Supplementary material for: Divergence of Gene Body DNA Methylation and Evolution of Plant Duplicate Genes
Source: PLoS One. 2014 Oct 13;9(10):e110357. doi: 10.1371/journal.pone.0110357 (PMC4195714; doi:10.1371/journal.pone.0110357)
Supplement: Table S5 — The correlation between methylation level and theta in Arabidopsis. (PDF) [file pone.0110357.s007.pdf]

Table S5. The correlation between methylation level and theta (θ) in *Arabidopsis*

	Spearman coefficients	*p* value	linear regression coefficient	coefficient significance	R-squared	smoothing spline	R-squared
Methylation level < 0.5						theta (θ)	0.1022
Theta (θ)	-0.0815	8.01E-08	-0.13318	3.67E-08	0.006984		
Methylation level ≥ 0.5							
Theta (θ)	0.5621	< 2.2e-16	1.10134	<2e-16	0.4304		
