# Supplementary material for: Divergence of Gene Body DNA Methylation and Evolution of Plant Duplicate Genes
Source: PLoS One. 2014 Oct 13;9(10):e110357. doi: 10.1371/journal.pone.0110357 (PMC4195714; doi:10.1371/journal.pone.0110357)
Supplement: Table S6 — The correlation between theta and other factors. (PDF) [file pone.0110357.s008.pdf]

Table S6. The correlation between theta (θ) and other factors

Factors	coeff	*p* value
24sRNA abundance ratio	0.2557668	< 2.2e-16
21sRNA abundance ratio	0.1307716	< 2.2e-16
Gene length	-0.1987435	< 2.2e-16
Exon number	-0.1250606	< 2.2e-16
Promoter methylation	0.2125488	< 2.2e-16
Gene body methylation >=0.5	0.6140651	< 2.2e-16
Gene body methy lation < 0.5	-0.09191964	< 2.2e-16
Expression	-0.1987	< 2.2e-16
