# Supplementary material for: Divergence of Gene Body DNA Methylation and Evolution of Plant Duplicate Genes
Source: PLoS One. 2014 Oct 13;9(10):e110357. doi: 10.1371/journal.pone.0110357 (PMC4195714; doi:10.1371/journal.pone.0110357)
Supplement: Table S7 — The relationship of methylation conservation and expression divergence that is corresponded to Figure 6 . (PDF) [file pone.0110357.s009.pdf]

Table S7. The relationship of methylation conservation and expression divergence which is corresponded to Figure 6

	Arabidopsis		
	Expression fold change		
Methylation level	CHM<CLM	CHM<NCM	CHM<CNM
	8.02E-09	2.29E-09	2.01E-08
Methylation pattern	CHM<CLM	CHM<NCM	CHM<CNM
	1.38E-05	0.002997	1.95E-05
	Expression specificity change		
Methylation level	CHM<CLM	CHM<NCM	CHM<CNM
	0.0251	0.0006662	0.01473
Methylation pattern	CHM<CLM	CHM<NCM	CHM<CNM
	0.003464	0.005339	0.007594
	**Rice**		
	Expression fold change		
Methylation level	CHM<CLM	CHM<NCM	CHM<CNM
	9.73E-06	1.37E-07	0.002199
Methylation pattern	CHM<CLM	CHM<NCM	CHM<CNM
	6.21E-09	3.97E-05	0.003855
