# Supplementary material for: Divergence of Gene Body DNA Methylation and Evolution of Plant Duplicate Genes
Source: PLoS One. 2014 Oct 13;9(10):e110357. doi: 10.1371/journal.pone.0110357 (PMC4195714; doi:10.1371/journal.pone.0110357)
Supplement: Table S8 — The relationship of methylation conservation and expression divergence with gene structure controlled. (PDF) [file pone.0110357.s010.pdf]

Table S8. The relationship of methylation conservation and expression divergence with gene structure controlled

**Rice**				
**CHM<CLM level**				
Gene length	p value of wilcox test of expression level changes	p value of wilcox test of gene length change	number of paralogs with CHM level	number of paralogs with CLM level
500~2500	0.0002194	0.169	43	163
**CHM<NCM level**				
Gene length	p value of wilcox test of expression level changes	p value of wilcox test of gene length change	number of paralogs with CHM level	number of paralogs with NCM level
3000~4000	4.10E-02	0.9105	50	50
**CHM<CLM pattern**				
Gene length	p value of wilcox test of expression level changes	p value of wilcox test of gene length change	number of paralogs with CHM pattern	number of paralogs with CLM pattern
500~2500	1.27E-04	0.2726	44	177
**CHM<NCM pattern**				
Gene length	p value of wilcox test of expression level changes	p value of wilcox test of gene length change	number of paralogs with CHM pattern	number of paralogs with NCM pattern
1500~3000	0.008185	0.4067	57	82
**CHM<CLM level**				
Exon number	p value of wilcox test of expression level changes	p value of wilcox test of exon number change	number of paralogs with CHM level	number of paralogs with CLM level
4~5	4.03E-04	0.7589	62	67
**CHM<NCM level**				
Exon number	p value of wilcox test of expression level changes	p value of wilcox test of exon number change	number of paralogs with CHM level	number of paralogs with NCM level
4~5	1.91E-03	0.705	62	96
**CHM<CLM pattern**				
Exon number	p value of wilcox test of expression level changes	p value of wilcox test of exon number change	number of paralogs with CHM pattern	number of paralogs with CLM pattern
4~5	3.91E-05	0.9491	65	78
**CHM<NCM pattern**				
Exon number	p value of wilcox test of expression level changes	p value of wilcox test of exon number change	number of paralogs with CHM pattern	number of paralogs with NCM pattern
4~6	0.0009098	0.5203	103	103
Arabidopsis				
**CHM<CLM level**				
Gene length	p value of wilcox test of expression level changes	p value of wilcox test of gene length change	number of paralogs with CHM level	number of paralogs with CLM level
1000~2000	0.03501	0.1477	61	222
**CHM<NCM level**				
Gene length	p value of wilcox test of expression level changes	p value of wilcox test of gene length change	number of paralogs with CHM level	number of paralogs with NCM level
1500~2500	0.009822	0.208	111	242
**CHM<CLM pattern**				
Gene length	p value of wilcox test of expression level changes	p value of wilcox test of gene length change	number of paralogs with CHM pattern	number of paralogs with CLM pattern
2500-4000	0.008754	0.221	200	81
**CHM<NCM pattern**				
Gene length	p value of wilcox test of expression level changes	p value of wilcox test of gene length change	number of paralogs with CHM pattern	number of paralogs with NCM pattern
2000-4000	0.007504	0.2385	315	244
**CHM<CLM level**				
Exon number	p value of wilcox test of expression level changes	p value of wilcox test of exon number change	number of paralogs with CHM level	number of paralogs with CLM level
2~4	0.03271	0.113	99	239
**CHM<NCM level**				
Exon number	p value of wilcox test of expression level changes	p value of wilcox test of exon number change	number of paralogs with CHM level	number of paralogs with NCM level
4~6	0.02283	0.7382	91	162
**CHM<CLM pattern**				
Exon number	p value of wilcox test of expression level changes	p value of wilcox test of exon number change	number of paralogs with CHM pattern	number of paralogs with CLM pattern
4~6	0.00753	0.3422	82	160
**CHM<NCM pattern**				
Exon number	p value of wilcox test of expression level changes	p value of wilcox test of exon number change	number of paralogs with CHM pattern	number of paralogs with NCM pattern
4~7	0.04678	0.5706	125	126
