# Supplementary material for: Divergence of Gene Body DNA Methylation and Evolution of Plant Duplicate Genes
Source: PLoS One. 2014 Oct 13;9(10):e110357. doi: 10.1371/journal.pone.0110357 (PMC4195714; doi:10.1371/journal.pone.0110357)
Supplement: Table S9 — The relationship of methylation conservation and expression divergence with nucleotide substitution rate controlled. (PDF) [file pone.0110357.s011.pdf]

Table S9. The relationship of methylation conservation and expression divergence with nucleotide substition rate controlled

		**Rice**		
	**CHM<CLM level**			
Substition rate	p value of wilcox test of **expression level** changes	p value of wilcox test of substition rate difference	number of paralogs with CHM level	number of paralogs with CLM level
0.05~0.25	0.02548	0.5714	137	131
	**CHM<NCM level**			
Substition rate	p value of wilcox test of **expression level** changes	p value of wilcox test of substition rate difference	number of paralogs with CHM level	number of paralogs with NCM level
0.05~0.25	0.003423	0.5377	137	167
	**CHM<CLM pattern**			
Substition rate	p value of wilcox test of **expression level** changes	p value of wilcox test of substition rate difference	number of paralogs with CHM pattern	number of paralogs with CLM pattern
0.05~0.25	8.14E-04	0.8509	148	141
	**CHM<NCM pattern**			
Substition rate	p value of wilcox test of **expression level** changes	p value of wilcox test of substition rate difference	number of paralogs with CHM pattern	number of paralogs with NCM pattern
0.05~0.25	0.002966	0.9877	148	116
		Arabidopsis		
	**CHM<CLM level**			
Substition rate	p value of wilcox test of **expression level** changes	p value of wilcox test of substition rate difference	number of paralogs with CHM level	number of paralogs with CLM level
0.05~0.2	0.04954	0.116	179	199
	**CHM<NCM level**			
Substition rate	p value of wilcox test of **expression level** changes	p value of wilcox test of substition rate difference	number of paralogs with CHM level	number of paralogs with NCM level
0.05-0.2	4.49E-03	0.8127	179	162
	**CHM<CLM pattern**			
Substition rate	p value of wilcox test of **expression level** changes	p value of wilcox test of substition rate difference	number of paralogs with CHM pattern	number of paralogs with CLM pattern
0.01-0.3	0.008743	0.1103	371	321
	**CHM<NCM pattern**			
Substition rate	p value of wilcox test of **expression level** changes	p value of wilcox test of substition rate difference	number of paralogs with CHM pattern	number of paralogs with NCM pattern
0.01-0.3	1.91E-02	0.1671	371	361
	**CHM<CLM level**			
Substition rate	p value of wilcox test of **expression specificity** changes	p value of wilcox test of substition rate difference	number of paralogs with CHM level	number of paralogs with CLM level
0.05-0.35	0.05418	0.9763	315	276
	**CHM<NCM level**			
Substition rate	p value of wilcox test of **expression specificity** changes	p value of wilcox test of substition rate difference	number of paralogs with CHM level	number of paralogs with NCM level
0.01~0.3	3.74E-03	0.4756	270	284
	**CHM<CLM pattern**			
Substition rate	p value of wilcox test of **expression specificity** changes	p value of wilcox test of substition rate difference	number of paralogs with CHM pattern	number of paralogs with CLM pattern
0.05~0.35	1.65E-02	0.6322	269	244
	**CHM<NCM pattern**			
Substition rate	p value of wilcox test of **expression specificity** changes	p value of wilcox test of substition rate difference	number of paralogs with CHM pattern	number of paralogs with NCM pattern
0.05~0.25	0.05397	0.4199	146	163
