# Supplementary material for: Divergence of Gene Body DNA Methylation and Evolution of Plant Duplicate Genes
Source: PLoS One. 2014 Oct 13;9(10):e110357. doi: 10.1371/journal.pone.0110357 (PMC4195714; doi:10.1371/journal.pone.0110357)
Supplement: Table S10 — The relationship of methylation conservation and expression divergence with small RNA abundance controlled. (PDF) [file pone.0110357.s012.pdf]

Table S10. The relationship of methylation conservation and expression divergence with small RNA abundance controlled

| | | | | |
|---|---|---|---|---|
| **Rice** | | | | |
| **expression level divergence** | **CHM<CLM level** | | | |
| p value of wilcox test of **expression level** changes | number of paralogs with CHM level | number of paralogs with CLM level | 24nt ratio | 21nt ratio |
| 1.01E-02 | 84 | 117 | 0.244 | 0.8987 |
| | **CHM<NCM level** | | 24nt ratio | 21nt ratio |
| p value of wilcox test of **expression level** changes | number of paralogs with CHM level | number of paralogs with NCM level | 0.2869 | 0.8688 |
| 5.16E-02 | 84 | 135 | | |
| | **CHM<CLM pattern** | | | |
| p value of wilcox test of **expression level** changes | number of paralogs with CHM pattern | number of paralogs with CLM pattern | 24nt ratio | 21nt ratio |
| 7.13E-04 | 98 | 110 | 0.618 | 0.8623 |
| | **CHM<NCM pattern** | | | |
| p value of wilcox test of **expression level** changes | number of paralogs with CHM pattern | number of paralogs with NCM pattern | 24nt ratio | 21nt ratio |
| 1.50E-01 | 98 | 103 | 0.5386 | 0.5558 |
| ***Arabidopsis*** | | | | |
| **expression level divergence** | **CHM<CLM level** | | | |
| p value of wilcox test of **expression level** changes | number of paralogs with CHM level | number of paralogs with CLM level | | |
| 3.95E-02 | 61 | 120 | | |
| | **CHM<NCM level** | | | |
| p value of wilcox test of **expression level** changes | number of paralogs with CHM level | number of paralogs with NCM level | | |
| 7.03E-03 | 61 | 94 | | |
| | **CHM<CLM pattern** | | | |
| p value of wilcox test of **expression level** changes | number of paralogs with CHM pattern | number of paralogs with CLM pattern | | |
| 0.1351 | 59 | 115 | | |
| | **CHM<NCM pattern** | | | |
| p value of wilcox test of **expression level** changes | number of paralogs with CHM pattern | number of paralogs with NCM pattern | | |
| 0.04273 | 59 | 48 | | |
