# Supplementary material for: Divergence of Gene Body DNA Methylation and Evolution of Plant Duplicate Genes
Source: PLoS One. 2014 Oct 13;9(10):e110357. doi: 10.1371/journal.pone.0110357 (PMC4195714; doi:10.1371/journal.pone.0110357)
Supplement: Table S11 — The correlation between exonic/intronic methylation divergence and expression level divergence. (PDF) [file pone.0110357.s013.pdf]

Table S11. The correlation between exonic/intronic methylation divergence and expression level divergence

Rice	coefficient correlation	*p* value
Exonic regions	-0.1371217	1.74E-05
Intronic regions	-0.1083941	0.005105
Arabidopsis	coefficient correlation	p-value
Exonic regions	-0.1767905	2.95E-10
Intronic regions	-0.1045024	0.07819
