# Supplementary material for: Divergence of Gene Body DNA Methylation and Evolution of Plant Duplicate Genes
Source: PLoS One. 2014 Oct 13;9(10):e110357. doi: 10.1371/journal.pone.0110357 (PMC4195714; doi:10.1371/journal.pone.0110357)
Supplement: Table S12 — The p-values corresponding to the different flanking region length cutoffs in searching nearby TEs. (PDF) [file pone.0110357.s014.pdf]

Table S12. The *p* values corresponding to the different flanking region length cutoffs in searching nearby TEs

| Flanking region | 100bp | 500bp | 1000bp | 1500bp | 2000bp |
|---|---|---|---|---|---|
| *p* value | 0.002195 | 0.02153 | 0.004606 | 0.03737 | 0.05308 |
